# Membrane translocation of t-SNARE protein syntaxin-4 abrogates ground-state pluripotency in mouse embryonic stem cells

**DOI:** 10.1038/srep39868

**Published:** 2017-01-06

**Authors:** Natsumi Hagiwara-Chatani, Kota Shirai, Takumi Kido, Tomoatsu Horigome, Akihiro Yasue, Naoki Adachi, Yohei Hirai

**Affiliations:** 1 Department of Biomedical Chemistry, Graduate School of Science and Technology, Kwansei Gakuin University, Sanda, Japan; 2 Department of Orthodontics and Dentofacial Orthopedics, Institute of Health Biosciences, The University of Tokushima, Tokushima, Japan

## Abstract

Embryonic stem (ES) and induced pluripotent stem (iPS) cells are attractive tools for regenerative medicine therapies. However, aberrant cell populations that display flattened morphology and lose ground-state pluripotency often appear spontaneously, unless glycogen synthase kinase 3β (GSK3β) and mitogen-activated protein kinase kinase (MEK1/2) are inactivated. Here, we show that membrane translocation of the t-SNARE protein syntaxin-4 possibly is involved in this phenomenon. We found that mouse ES cells cultured without GSK3β/MEK1/2 inhibitors (2i) spontaneously extrude syntaxin-4 at the cell surface and that artificial expression of cell surface syntaxin-4 induces appreciable morphological changes and mesodermal differentiation through dephosphorylation of Akt. Transcriptome analyses revealed several candidate elements responsible for this, specifically, an E-to P-cadherin switch and a marked downregulation of Zscan4 proteins, which are DNA-binding proteins essential for ES cell pluripotency. Embryonic carcinoma cell lines F9 and P19CL6, which maintain undifferentiated states independently of Zscan4 proteins, exhibited similar cellular behaviors upon stimulation with cell surface syntaxin-4. The functional ablation of E-cadherin and overexpression of P-cadherin reproduced syntaxin-4-induced cell morphology, demonstrating that the E- to P-cadherin switch executes morphological signals from cell surface syntaxin-4. Thus, spontaneous membrane translocation of syntaxin-4 emerged as a critical element for maintenance of the stem-cell niche.

Embryonic stem (ES) cells cultured without inhibitors of glycogen synthase kinase 3β (GSK3β) and mitogen-activated protein kinase kinase (MEK1/2) (2i) are prone to exhibit heterogeneity in morphology and in the ground state of pluripotency^1^. Spontaneous generation of such aberrant cells cannot be completely prevented even in the presence of leukemia inhibitory factor (LIF), which supports the expression of the stemness factor Oct3/4 by activating either Jak-Stat3 or phosphoinositide 3-kinase (PI3K)/Akt signaling pathways^2^. Previous studies have demonstrated that Nanog and Rex1, other transcription factors involved in pluripotency, asynchronously fluctuate over time in clonal ES cells; this might affect the differentiation potential of each ES cell in the same colony^3^,^4^,^5^. For example, *nanog*-negative ES cells tend to differentiate into primitive endoderm-like cells^6^ and *Rex1*-negative cell populations behave like epiblasts or primitive ectoderm cells, and more readily differentiate into somatic lineages^7^. In addition, the cyclic gene *Hes1* is reportedly asynchronously expressed in clonal ES cells, and contributes to heterogeneous responses to differentiation stimuli; low or high expression levels result in differentiation into neural or mesodermal cells, respectively^8^,^9^. The up- and downregulation of these cellular context determinants are always accompanied by dramatic morphological alternations, and conversely, forced cell shape changes could act as differentiation cues^10^. This suggests a close connection between morphology and differentiation potential in stem cells.

The decrease in ES cell clonogenic capacity is considered a consequence of temporal epithelial to mesenchymal-transition (EMT)^11^,^12^. EMT is characterized by downregulation of E-cadherin, a target of transcriptional repression by snail family proteins, and upregulation of N-cadherin^13^,^14^. E-cadherin downregulation is closely linked to inactivation of PI3K/Akt signals that regulates GSK3β, a key element of the Wnt/β-catenin signaling pathway in ES cells^15^. Thus, the functional modulation of these key elements is crucial for ES cell stabilization^16^,^17^. In addition, considerable attention has been given to P-cadherin, an alternative cadherin that is upregulated during EMT. Increased expression of P-cadherin has been detected in many cancer cells^18^, and P-cadherin appears to promote aggressive/invasive properties in several tumor cells^19^. Zscan4 family members have recently emerged as key factors that maintain ground-state pluripotency. These factors appear to play critical roles in the stability/integrity of the ES cell genome, and functional knockdown of cognate Zscan4 members resulted in decreased self-renewal potential in ES cells^20^,^21^. The Zscan4 family comprises six paralogs (Zscan4a-f) and three pseudogenes, the expression of which has been specifically detected in 2-cell embryos *in vivo* and in ES cells *in vitro*^22^,^23^. The expression of these proteins is also regulated by PI3K/Akt, suggesting the importance of this pathway for maintaining the ground state of pluripotency in ES cells^23^. Given the spontaneous appearance of aberrant cell populations, with altered morphology and differentiation potential, in clonal stem cell colonies, one might predict the existence of unidentified non-diffusible extracellular signals that can focally and temporally regulate the PI3K/Akt pathway to maintain stem cell plasticity.

Syntaxin family proteins are membrane-tethered proteins that mediate intracellular vesicle fusions as t-SNARE components^24^,^25^. However, subpopulations of certain plasmalemmal proteins, including syntaxin2 (epimorphin) and syntaxin-4, reportedly translocate across the cell membrane to locally act as non-diffusible signaling molecules^26^,^27^,^28^,^29^,^30^,^31^. For example, certain cells of the embryonic carcinoma (EC) cell line F9 extrude syntaxin-4 as membrane-tethered proteins to locally affect the morphology, motility, and cellular context of adjacent cells^27^.

In the present study, we detected local extrusion of syntaxin-4 in ES cell colonies even in the absence of differentiation stimuli. Using an inducible expression system, we found that extracellular expression of syntaxin-4 leads to dephosphorylation of Akt, morphological changes, an E- to P-cadherin switch, and the early onset of differentiation, with downregulation of Zscan4 genes, in ES cells. Thus, local membrane translocation of syntaxin-4 has emerged as a key element for stem-cell plasticity.

## Results

### Expression of syntaxin-4 in mouse ES cells

**Figure 1:**
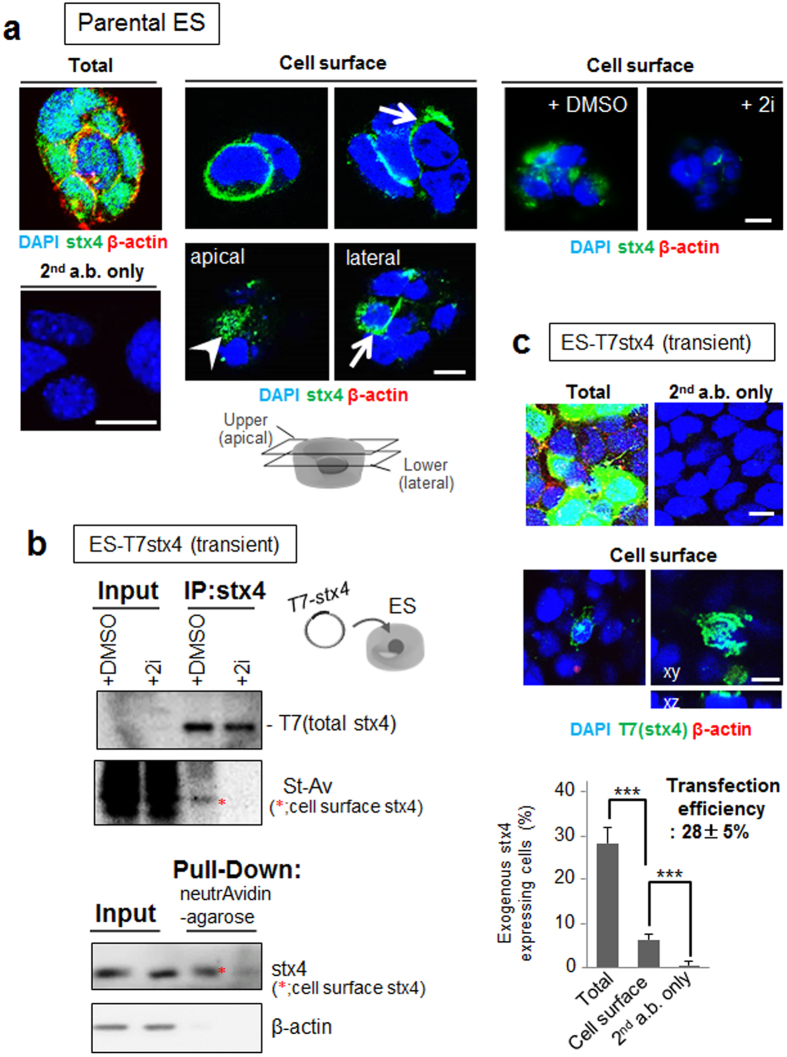
Spontaneous expression of syntaxin-4 at the surface of ES cells. (**a**), Endogenous syntaxin-4 (stx4; green) was detected with anti-syntaxin-4 antibodies in ES cells before (Cell surface) and after membrane permeabilization (Total). The cells were precultured in the presence (+2i) or absence (−2i) of GSK3β/MEK1/2 inhibitors for two days. As an internal control, cytoplasmic β-actin was simultaneously detected (red). Left lower column, cells were stained only with secondary antibodies (2^nd^ a.b.). All cells abundantly express β-actin and syntaxin-4 (left). Whereas a small population of the cells cultured in the absence of 2i display cell surface syntaxin-4 at the lateral (arrows) and apical (arrowhead) membrane, cells treated with 2i apparently decrease the extrusion of syntaxin-4 at the cell surface (right images). DMSO alone was used as a control (right images). Cell nuclei were counterstained with DAPI. Bars, 10 μm. (**b**), ES cells were transfected with syntaxin-4 tagged only with T7 peptide (T7stx4) and surface expression of exogenous syntaxin-4 in cells cultured with (+2i) or without (+DMSO) 2i for two days was analyzed. Upper panel, cell surface proteins were labeled with NHS-sulfo-biotin, and the cell lysate (Input) or immunoprecipitates with anti-syntaxin-4 antibodies (IP:stx4) was analyzed with HRP-labeled anti-T7 antibody (T7) or HRP-labeled streptavidin (St-Av). Lower panel, biotinylated cell surface proteins in the cell lysate (Input) or those retrieved with neutrAvidin-beads (Pull-Down) were analyzed with antibodies against syntaxin-4 or cytoplasmic β-actin. Cropped areas from the original blots (Supplementary Fig. S1) are shown. Treatment with 2i clearly decreases the cell surface expression of T7 tagged-syntaxin-4 (*). (**c**), ES cells were transfected with T7 tagged syntaxin-4 (T7stx4), and exogenous syntaxin-4 was labeled with anti-T7 tag antibodies (green) after 24 h. As an internal control, cytoplasmic β-actin was simultaneously stained (red). Upper images, anti-T7 tag antibody clearly detects the syntaxin-4 transgene product, but not β-actin, at the cell surface. Bars, 10 μm. Lower panel, the ratio of cells exhibiting syntaxin-4 at the cell surface in transfected cells. Approximately 20% of T7stx4-expressing cells (transfection efficiency, 28 ± 5%) extrudes T7stx4 at the cell surface. N = 18, ***p < 0.001.

In certain cell types, a subpopulation reportedly extruded extracellular syntaxin-4 to locally propagate signals for morphogenesis and differentiation^27^,^30^. To determine whether this is also the case in ES cells, extracellular exposure to endogenous syntaxin-4 was investigated in cells grown in LIF-containing medium. GSK3β/MEK1/2, key elements of Wnt/mitogen-activated protein kinase (MAPK) signaling, have been proposed to play regulatory roles in ES cell stemness, and the simultaneous inhibition of these signaling pathways resulted in the maintenance of ES cells in the ground state of pluripotency^1^. We found that all cells expressed cytoplasmic syntaxin-4; however, certain cell populations extruded syntaxin-4 at lateral and apical cell surfaces when cultured without GSK3β/MEK1/2 inhibitors (2i) (Fig. 1a). The effect of 2i on the membrane translocation of syntaxin-4 was confirmed in lysate of ES cells transfected with a T7 tagged-syntaxin-4 transgene (T7stx4) (Fig. 1b and Supplementary Fig. S1). The ratio of cells presenting extracellular syntaxin-4 was approximately 20%, which was confirmed in the transfected ES cells (Fig. 1c). These results demonstrated that clonal stem cells extrude syntaxin-4 in the absence of potent pluripotency sustaining reagents.

### Extracellular syntaxin-4 results in morphological changes and mesodermal differentiation in ES cells

**Figure 2:**
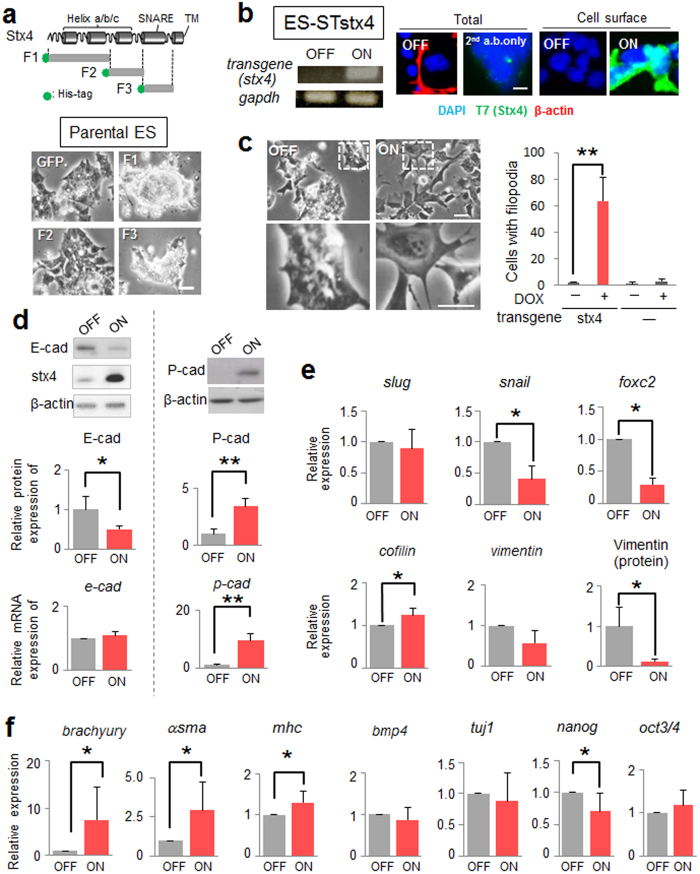
Effect of cell surface expression of syntaxin-4 on ES cell behavior. (**a**), Upper, schematic diagram of syntaxin-4 and 6X histidine-tagged recombinant fragments F1 (Met1–Glu110), F2 (Ala111–Arg197), and F3 (Gln198–Lys272). F1 contains N-terminal helices a and b, F2 contains helix c and F3 contains the SNARE domain. Lower, phase-contrast images of ES cells incubated with recombinant F1, F2, F3 or GFP control for three days. Bar, 20 μm. (**b**), The inducible expression of exogenous syntaxin-4 in ES-STstx4 was analyzed by RT-PCR (left) and immunocytochemistry using antibodies against β-actin and the T7 tag (right). Bar, 10 μm. (**c**), Left panels, appreciable phenotypic changes are induced by the cell surface expression of syntaxin-4 (ON) for 48 h. Lower, large images of upper insets. Bars, 20 μm. Right, Quantification of cells with active pseudopodial protrusions. N = 4, ** *p* < 0.01. (**d**), Cells were analyzed for the expression of E-cadherin (E-cad) or P-cadherin (P-cad) at the protein and mRNA level. The representative blots (upper panels) and the quantitation of the expression relative to that of β-actin (middle and lower panels) are shown. For E-cadherin, N = 4, *p < 0.05. For P-cadherin, N = 3, **p < 0.01. For upper panels, cropped areas from the original blots (Supplementary Fig. S1) are shown. (**e**), The canonical EMT marker *slug* is unchanged while the other markers *snail* and *foxc2* are downregulated. The cytoskeletal component vimentin is decreased, whereas the regulator of actin dynamics *cofilin* is significantly increased, albeit to a lesser extent. N = 4, * *p* < 0.05. (**f**), Cell surface expression of syntaxin-4 leads to a clear upregulation of *brachyury, α-smooth muscle actin (αsma),* and *myosin heavy chain (mhc)*, while the expression of *bmp4* and *tuj1* is unchanged. N = 3, *p < 0.05. The stem cell marker *nanog* is downregulated, whereas the marker *oct3/4* remains unchanged. N = 4, *p < 0.05.

To know the possible role of extruded syntaxin-4, we tested an antagonistic effect of the recombinant syntaxin-4 fragment prepared in the previous study^32^. As was the case in mammary epithelial cells, the recombinant protein of the SNARE domain (F3), but not of the helix c (F2) gave effect on ES cell morphology; ES cells formed more densely packed–colonies, indicative of restoration of the stemness (Fig. 2a). Intriguingly, a fragment of the N-terminal domain of syntaxin-4 (F1) also showed the effect similar to F3 (Fig. 2a), and this fragment clearly reduced the expression of a downstream element of cell surface syntaxin-4 in ES cells (see below, and Supplementary Fig. S2), indicating a critical role of the membrane translocation of syntaxin-4 in ES cell behaviors. To define the role of membrane-tethered extracellular syntaxin-4, we generated ES cells with inducible expression of cell surface syntaxin-4 (STstx4) using a PiggyBac transposon inducible expression system^33^,^34^. The transposon carried a cDNA encoding syntaxin-4 connected to cDNAs for a IL-2 signal peptide and T7-tag for extracellular localization and effective detection, respectively^27^. Cells expressed T7-tagged syntaxin-4 at the cell surface upon treatment with doxycycline (Dox) (Fig. 2b). In response to inducible expression of cell surface syntaxin-4, these cells lost their cell–cell adhesive property and showed an altered morphology, exhibiting active filopodial protrusions, after a few days, even in the presence of LIF (Fig. 2c). Such cell behaviors are indicative of EMT, which reportedly precedes cyto-differentiation in ES cells^11^,^12^. Parental ES cells without the syntaxin-4 transgene did not show similar morphological changes upon treatment with Dox (Fig. 2c). We found that ES cells with extracellular syntaxin-4 downregulated E-cadherin, a major mediator of epithelial cell-cell adhesion; however, its mRNA level was unchanged (Fig. 2d). Instead, these cells expressed P-cadherin, which has been shown to temporally increase in several invasive tumors to promote cellular mobility^19^ (Fig. 2d), implying that an E- to P-cadherin switch might execute signals propagated by syntaxin-4. It is noteworthy that the expression of *Snail* and *Slug,* canonical transcription factors involved in tumorigenic transformation, was not increased, while the fibroblastic marker vimentin was apparently downregulated (Fig. 2e). However, these cells upregulated mesodermal markers including *brachyury,* a critical T-box transcription factor involved in gastrulation/mesodermal differentiation, α-*smooth muscle actin*, and *myosin heavy chain* (Fig. 2f). The stemness marker *nanog* was downregulated, whereas the *oct3/4* level remained unchanged (Fig. 2f). We could exclude the possibility that the EMT-like cell behaviors were instructed as a consequence of the artificial gene manipulation; the phenotypical features induced by cell surface expression of syntaxin-4 were clearly receded when the antagonistic fragment of syntaxin-4 (F1) was present in the medium (Supplementary Fig. S2).

### Identification of other downstream mediators of cell surface syntaxin-4

**Figure 3:**
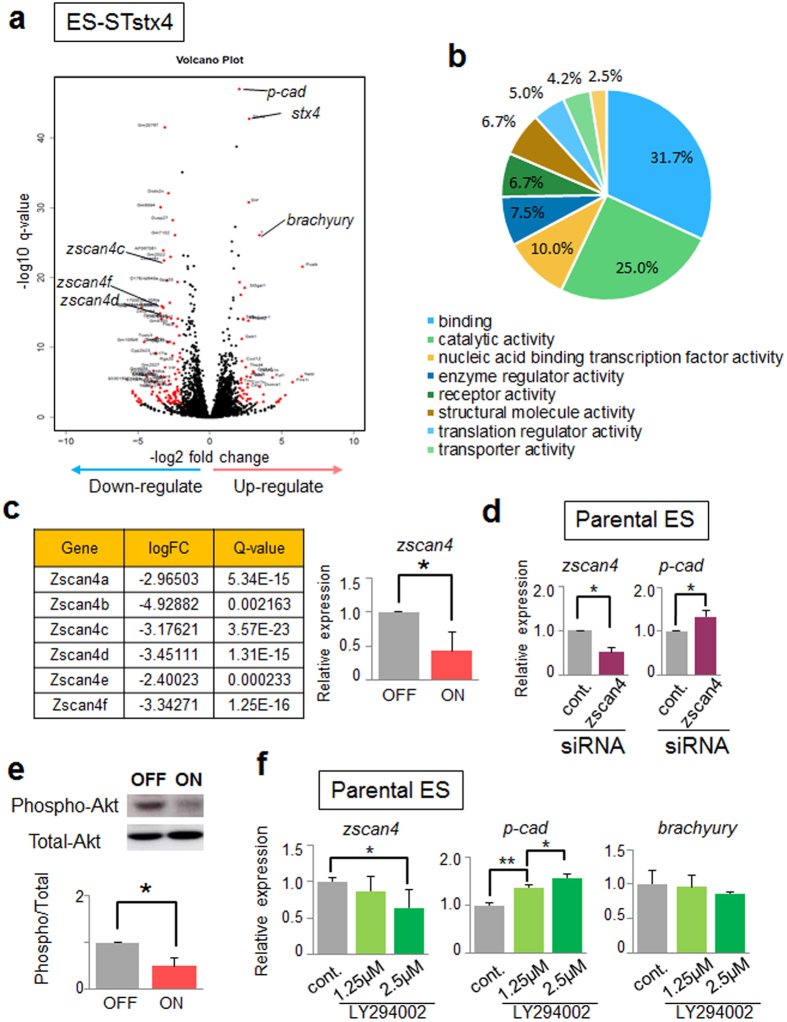
Comprehensive analysis of elements downstream of cell surface syntaxin-4. (**a**), Volcano plot showing DE genes in ES-STstx4 cells. DE genes that were markedly up- and downregulated in syntaxin-4-ON samples with an absolute LogFC value ≥2 and a q-value <0.01 are highlighted in red (157 genes). (**b**), Pie chart of molecular functions of up/downregulated genes. (**c**), A striking reduction in *zscan4* by extracellular syntaxin-4 is observed. Left, list of LogFC and Q-values for all Zscan4 members. Right, qRT-PCR analysis of expression of cognate *zscan4* in cells with (ON) and without (OFF) cell surface syntaxin-4. The primer pair (Supplementary Table S2) can recognize *zscan4a, b, c, d,* and *f*. N = 3, *p < 0.05. (**d**), *p-cadherin* expression is significantly suppressed by siRNA-mediated knockdown of Zscan4 genes in parental ES cells. N = 4, *p < 0.05. (**e**) Phosphorylation of Akt in ES-STstx4 cells is reduced in cells with syntaxin-4 signaling (ON). N = 3, *p < 0.05. For upper panels, cropped areas from the original blots (Supplementary Fig. S1) are shown. (**f**), LY294002, an inhibitor of the PI3K/Akt signaling pathway, appears to upregulate *p-cadherin* and downregulate *zscan4s* in parental ES cells as observed in syntaxin-4-expressing ES cells. By contrast, the expression of *brachyury* is not affected. N = 3, *p < 0.05, **p < 0.01.

Next, we comprehensively analyzed factors downstream of cell surface syntaxin-4 that could mediate morphogenic and/or differentiation signals. Among the genes whose transcripts were up- or downregulated in ES-STstx4 cells, we selected 157 genes with LogFC absolute values ≥2, and q-values <0.01 (Fig. 3a, highlighted in red); this subset contained 19 genes whose expression was also changed in parental ES cells upon Dox treatment. Among thus selected 138 genes, 119 genes could be classified into several categories, using the PANTHER-GO-Slim system (Fig. 3b and Supplementary Table S1). In addition to the dramatic upregulation of *p-cadherin*, we detected marked downregulation of all members of the *zscan4* family (Fig. 3a,c), transient expression of which reportedly plays key roles in genomic stability, which is important for restoring ES cell pluripotency^21^. Upon siRNA–mediated silencing of the Zscan4 genes, *p-cadherin* expression was decreased in parental ES cells, albeit to a lesser extent than upon overexpression of syntaxin-4 (Fig. 2d), suggesting that these genes partly mediate the syntaxin-4 signals (Fig. 3d). These Zscan4 genes are known to be regulated by the PI3K/Akt pathway^23^; we found a significant reduction in Akt phosphorylation upon stimulation with syntaxin-4 (Fig. 3e). Notably, LY294002, an inhibitor of the PI3K/Akt pathway, suppressed the expression of *zscan4* transcription and promoted that of *p-cadherin* in parental ES cells (Fig. 3f). However, this reagent showed significant toxicity and had little effect on the expression of *brachyury* (Fig. 3f). These results suggested that syntaxin-4 extruded at the cell surface affects the PI3K/Akt signaling cascade to elicit cell responses, in which Zscan4 genes play an important role, even in the presence of LIF.

### Inhibition of GSK3β/MEK1/2 signals cannot completely abrogate functions of forcibly expressed cell surface syntaxin-4

**Figure 4:**
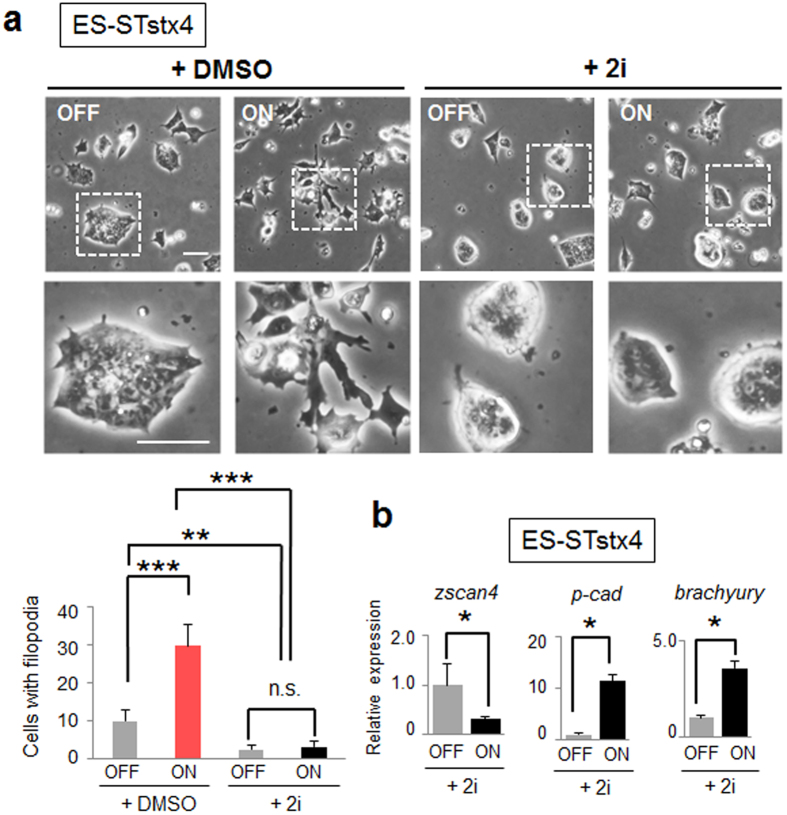
Inactivation of GSK3β/MEK1/2 signaling is not sufficient to block the effects of forcibly expressed cell surface syntaxin-4. (**a**), Morphological changes by cell surface syntaxin-4 (ON) are apparently inhibited by PD0325901 and CHIR99021 (2i), inhibitors of GSK3β and MEK1/2, respectively. Bars, 50 μm. The lower photographs represent enlarged images of the insets of the upper images. Lower graph, quantification of cells with filopodia. N = 3, **p < 0.01, ***p < 0.001, n.s., not significant. (**b**), Syntaxin-4-triggered changes in the expression of *p-cadherin, zscan4*, and *brachyury* are not affected by 2i. N = 3, *p < 0.05.

Similar to the antagonistic peptide of syntaxin-4, we found that 2i apparently blocked the morphological changes induced by forcible expression of cell surface syntaxin-4 (Fig. 4a). However, syntaxin-4-induced changes in *p-cadherin, zscan4,* and *brachyury* expression were not inhibited (Fig. 4b). These results suggested that whereas inhibitors of GSK3β/MEK1/2 blocked extracellular extrusion of endogenous syntaxin-4 (Fig. 1b), they were not able to abrogate differentiation initiation triggered by forcibly expressed cell surface syntaxin-4.

### Extracellular syntaxin-4 leads to an E- to P-cadherin switch and morphological changes in F9 cells

**Figure 5:**
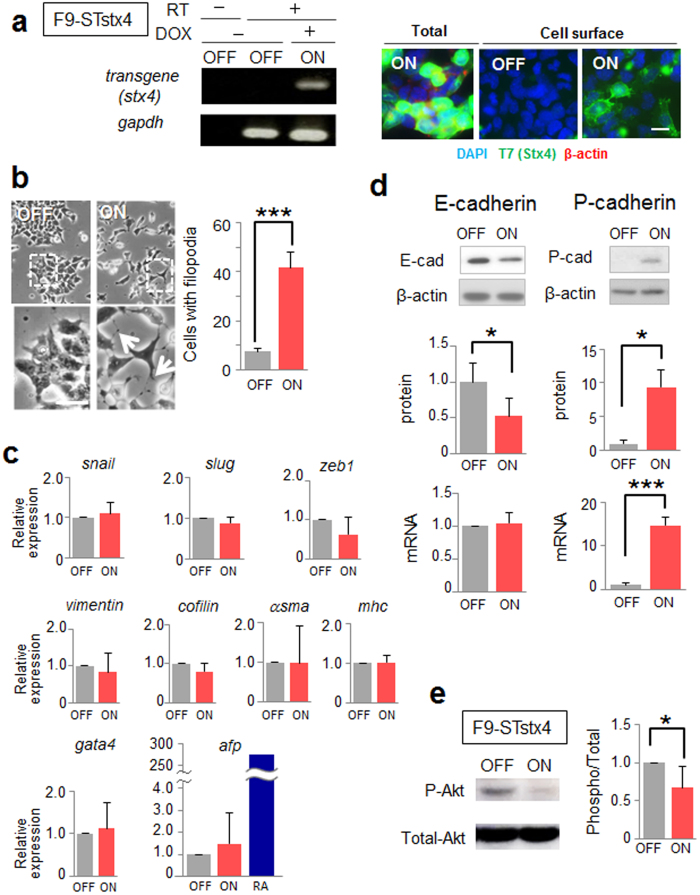
The effect of extracellular syntaxin-4 on F9 cell behaviors. (**a**), Analyses of inducible expression of exogenous syntaxin-4 in F9-STstx4 with qRT-PCR (left) and immunocytochemistry (right) after 72-h induction (ON). RT -, performed without reverse transcription. Bar, 10 μm. (**b**), Left images, appreciable phenotypic changes are induced by the cell surface expression of syntaxin-4 (ON) for 48 h. Lower images represent enlarged images of the upper insets. Bar, 20 μm. Right, quantification of cells with pseudopodial protrusions. N = 4, ***p < 0.001. Upon cell surface expression of syntaxin-4 (ON), the cells dissociate and display active filopodia. (**c**), The expression of all of canonical EMT markers *snail, slug*, and *zeb1*, cytoskeletal components *vimentin* and *cofilin,* mesodermal markers *α-smooth muscle actin (αsma*) and *myosin heavy chain (mhc*), and endodermal markers *gata4* and *α-fetoprotein (afp*) is unaffected by 48-h stimulation with extracellular syntaxin-4. The expression of all of the differentiation markers is not significantly different between “ON” and “OFF” samples. N = 4, *p < 0.05. For *afp,* upregulation by retinoic acid (RA; a positive control) is included. (**d**), Cells were analyzed for the expression of E-cadherin (E-cad) or P-cadherin (P-cad) at the protein and mRNA level. The representative blots (upper panels) and the quantitation of the amounts relative to that of β-actin (middle and lower panels) are shown. E-cadherin protein is significantly downregulated upon the induction of cell surface syntaxin-4, whereas mRNA is unaffected. N = 4, *p < 0.05. For upper panels, cropped areas from the original blots (Supplementary Fig. S1) are shown. (**e**), Phosphorylation of Akt is reduced in F9 cells upon stimulation with syntaxin-4. N = 3, *p < 0.05.

To define the role of extruded syntaxin-4 in ES cell behavior, we used the simpler, stable embryonic carcinoma (EC) F9 stem cell system, in which ES cell–specific regulators of differentiation, including a set of Zscan4 proteins, are undetectable (Supplementary Fig. S3). The F9 cell line expresses E-cadherin and forms compact colonies similar to ES cells, though these cells have lost their potential to differentiate into mesodermal cell lineages. F9 cells possessing the syntaxin-4 transgene (F9-STstx4) showed morphological responses similar to those of ES cells upon expression of cell surface syntaxin-4. Specifically, they lost cell–cell adhesiveness and showed a dramatically changed cell morphology, with active filopodial protrusions after a few days (Fig. 5a,b). However, significant changes in the expression of various differentiation markers, including those for canonical EMT (*snail, slug,* and *zeb1*), endodermal differentiation (*gata4* and *afp*), and fibroblastic cell types (*vimentin, cofilin, α-smooth muscle actin* and *myosin heavy chain*) were not evident in this short period (Fig. 5c). We found that the E- to P-cadherin switch, accompanied by inactivation of Akt signaling, was induced by cell surface expression of syntaxin-4 in F9 cells, as was the case in ES cells (Fig. 5d,e).

### E- to P-cadherin switch as a key element to mediate signals from syntaxin-4

**Figure 6:**
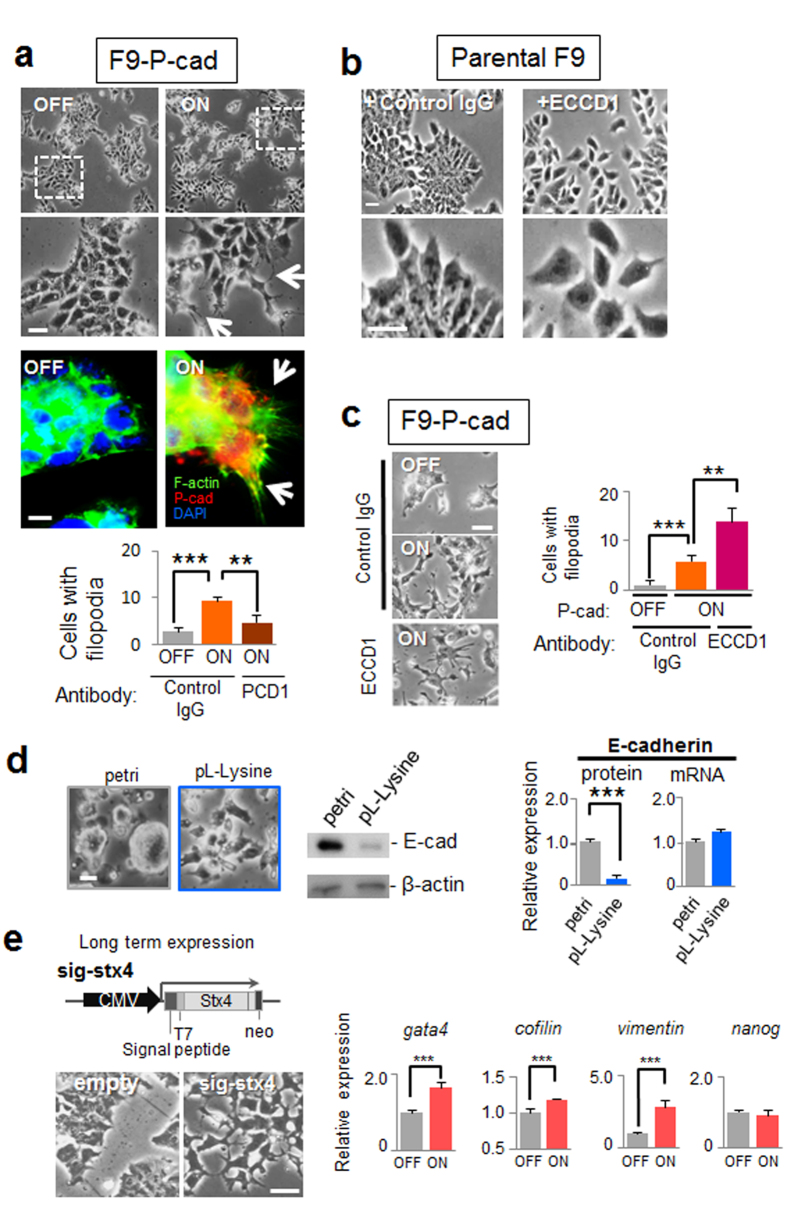
Effect of E- to P-cadherin switch on F9 cell morphology. (**a**), Upper panels, phase-contrast images of F9-P-cad cells with (ON) or without (OFF) expression of exogenous P-cadherin. Middle panels, P-cadherin (red) and F-actin (green) are visualized. Arrows, pseudopodial protrusions in cells with P-cadherin. Bars, 20 μm. Lower panels, quantification of cells with filopodia. Expression of exogenous P-cadherin leads to the formation of active filopodia, which is substantially blocked by the addition of an anti-P-cadherin antibody PCD1 (50 μg/ml). The number of active pseudopodia increases in P-cadherin-expressing cells. N = 4, **p < 0.01, ***p < 0.001. (**b**), Effect of E-cadherin blocking antibody (ECCD1) on parental F9 cell behavior. Treatment with ECCD1 causes the dissociation of cells without active pseudopodial protrusions. Bars, 20 μm. (**c**), F9-P-cad cells at the periphery of the colony extend filopodia in response to P-cadherin expression (ON), and the number of cells with filopodia increases when cells are simultaneously dissociated by ECCD1. Left panels, phase-contrast images. Bar, 20 μm. Right panel, quantification of cells with filopodia. N = 4, **p < 0.01, ***p < 0.001. (**d**), Substrate-adhesion affects E-cadherin expression at the protein level. Parental F9 cells adhering to and spreading on poly-l-lysine (PL-Lysine) show significantly decreased expression of E-cadherin (E-cad) protein, but not mRNA, compared to that of similar cells found in clusters on the Petri dish (petri). N = 3, ***p < 0.001. For middle panels, cropped areas from the original blots (Supplementary Fig. S) are shown. (**e**), Phenotypic appearance and expression profile of several markers in F9 cells with sustained expression of cell surface syntaxin-4. Left upper panel, schematic diagram of the construct used for sustained expression of cell surface syntaxin-4^27^. Lower panels, phase-contrast images of the cells with (sin-stx4) and without (empty) long-term stimulation with the transgene. Bar, 20 μm. Right, the expression of *gata4, cofilin,* and *vimentin* is increased, whereas that of the stemness marker *nanog* is unchanged. N = 4, ***p < 0.001.

To examine the association between the E- to P-cadherin switch and morphological changes, parental F9 cells were transfected with a P-cadherin transgene and/or treated with an anti-E-cadherin blocking antibody ECCD1. We found that cells over-expressing P-cadherin exhibited active pseudopodium formation, similar to that observed in cells stimulated with extracellular syntaxin-4, which was substantially blocked by anti-P-cadherin antibody PCD1, confirming the causal role of P-cadherin in the morphological alternation (Fig. 6a). On the other hand, cell–cell adhesion remained almost intact in cells with and without forcible expression of P-cadherin (Fig. 6a). In contrast, treatment with ECCD1 led to cellular dissociation without morphological changes (Fig. 6b), as reported previously^35^. We found that simultaneous over-expression of P-cadherin and functional inhibition of E-cadherin resulted in a phenotype quite similar to that observed with cell surface syntaxin-4 expression, revealing that the E- to P-cadherin switch mediates morphological changes associated with extracellular syntaxin-4 (Fig. 6c). However, we could not exclude the possibility that the decline in E-cadherin expression was merely due to syntaxin-4-induced changes to the cellular context since the expression of E-cadherin was apparently altered solely by modification of the substrate adhesive property of F9 cells; cells adherent to the substrate (using poly-l-lysine coat) showed dramatically decreased *E-cadherin* expression as compared to floating cells on petri-dishes (Fig. 6d). We could not assess the impact on differentiation in F9 cells as they do not readily differentiate into mesodermal lineages^36^. In addition, these cells succumb to apoptosis after long-term treatment with Dox. However, with sustained expression of cell surface syntaxin-4 using another expression construct^27^, F9 cells upregulated *gata4*, which is a transcription factor that acts as an endodermal differentiation cue in this cell type (Fig. 6e). In these cells, the levels of *cofilin*, a molecular regulator for actin dynamics^37^, and *vimentin*, encoding an intermediate filament in fibroblasts^38^, were also increased (Fig. 6e), suggesting that long-term signals propagated by extracellular syntaxin-4 affect differentiation even in non-mesodermal lineages.

### Extracellular syntaxin-4 induces mesodermal differentiation in P19CL6 cells

**Figure 7:**
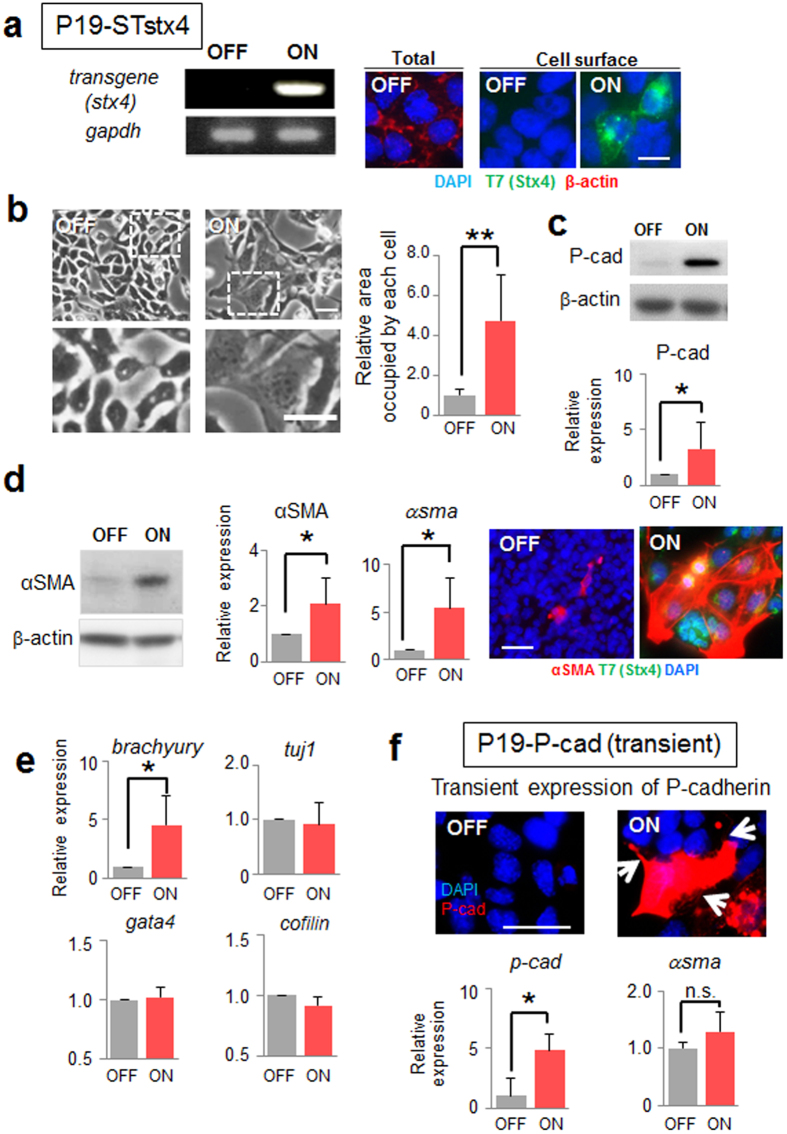
Effect of cell surface expression of syntaxin-4 on P19CL6 cell behavior. (**a**), The cell surface expression of syntaxin-4 transgene in P19-STstx4 was investigated with RT-PCR (left) and immunocytochemistry (right) after 72-h transgene induction. Bar, 10 μm. **(b**), Left, cells displaying flattened morphology upon cell surface expression of syntaxin-4 (ON). Lower panels, enlarged images of insets in upper images. Bars, 20 μm. Right panel, quantification of the relative area occupied by each cell. N = 10, **p < 0.01. (**c**), The expression of P-cadherin is increased also in this cell type in response to the expression of syntaxin-4 (upper). The representative blots cropped from the original blots (Supplementary Fig. S1) are shown. Lower panel, the amount of P-cadherin relative to that of β-actin. N = 3, *p < 0.05. (**d**), The mesodermal marker αSMA is upregulated by cell surface syntaxin-4 (ON). Left, representative blots cropped from the original blots (Supplementary Fig. S1) are shown. Middle, quantification of the expression of αSMA protein and mRNA relative to those of β-actin. N = 4, *p < 0.05. Right, expression of αSMA (red) and T7-syntaxin-4 (green) in cells with (ON) and without (OFF) cell surface syntaxin-4. Bar, 20 μm. (**e**), Expression of several differentiation markers, including the mesodermal marker *brachyury*, the ectodermal marker *tuji1*, the endodermal marker *gata4*, and the actin dynamics regulator *cofilin*. N = 4 (for *cofilin*, N = 3), *p < 0.05. P19CL6 cells with a flattened morphology resulting from cell surface syntaxin-4 differentiate into mesodermal lineages. (**f**), Transient expression of exogenous P-cadherin (red in upper images) in P19CL6 cells leads to flattened cell morphology with active filopodia (arrows), whereas the differentiation marker *αsma* is unchanged (lower). Bar, 20 μm. N = 3, *p < 0.05.

Finally, we tested the effects of cell surface expression of syntaxin-4 in another EC cell line, P19CL6, which retains the intrinsic potential to differentiate into mesodermal lineages^39^ (Fig. 7a). Upon induction of syntaxin-4, P19CL6 cells elicited similar morphogenetic responses and displayed a flattened fibroblastic morphology (Fig. 7b). Although they do not express detectable E-cadherin, cell surface syntaxin-4 led to the marked upregulation of P-cadherin also in this cell type (Fig. 7c). Whereas the mesodermal markers *α-smooth muscle actin* and *brachyury* were upregulated, the neuronal marker *tuj1* and cardiac marker *gata4* were unchanged (Fig. 7d,e). When P-cadherin was forcibly expressed, parental P19CL6 cells exhibited a morphology similar to that seen with cell surface syntaxin-4, whereas differentiation markers remained unchanged (Fig. 7f). It is noteworthy that exogenous P-cadherin did not accumulate at the cell–cell contact sites in both F9 and P19CL6 cells (Figs 6a and 7f).

**Figure 8:**
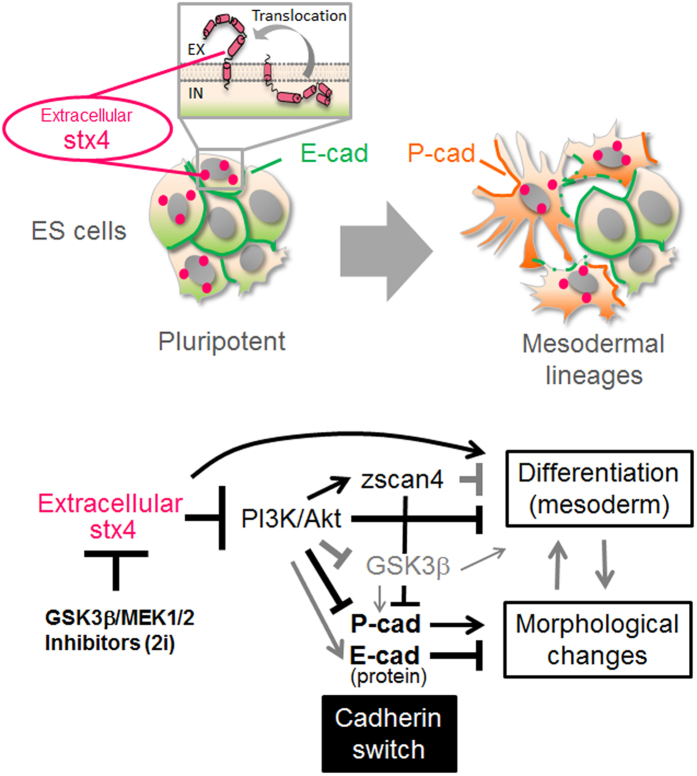
A plausible mechanism of the spontaneous loss of stemness in ES cells. Upper panel, a subpopulation of ES cells growing in a tightly packed colony that spontaneously extrude syntaxin-4, a membrane-tethered t-SNARE protein, which locally executes its latent extracellular functions. Lower panel, cell surface syntaxin-4 expressed in response to removal of 2i inactivates PI3K/Akt signaling, leading to an E- to P-cadherin switch for morphological changes and downregulation of stemness factors, such as cognate Zscan4 proteins, for the onset of differentiation. GSK3β, a negatively regulated target of PI3K/Akt, might be involved in the morphological changes. Bold lines, revealed by this study. Gray lines, suggested by previous studies.

Taken together, syntaxin-4, which is spontaneously extruded at the cell surface in response to the removal of stemness reagents 2i might play a critical role in the abrogation of ground-state pluripotency in ES cells, through inactivation of the PI3K/Akt signaling, induction of an E- to P-cadherin switch, and downregulation of several stemness factors including the Zscas4 family (Fig. 8).

## Discussion

Here, we provide evidence that syntaxin-4 spontaneously translocates across the cell membrane to execute its latent extracellular functions, including morphological changes and the onset of differentiation in ES cells; this leads to instability in ES cell stemness. As this effect does not require additional differentiation stimuli and is achieved even in the presence of LIF, the molecular mechanisms of syntaxin-4 membrane translocation are unclear at present. However, given that 2i inhibited cell surface expression of syntaxin-4, upstream extracellular elements of GSK3β or MEK1/2 might be involved in the extracellular extrusion of syntaxin-4. Alternatively, recent studies have revealed that epimorphin (syntaxin-2), another plasmalemmal syntaxin, is extruded as a component of synaptotagmin and annexin II protein complexes in response to apoptotic signals^40^. Thus, one could assume that the extracellular presentation of syntaxin-4 is a result of the cell competition in ES cell colonies, which spontaneously induces local apoptosis to eliminate unfit cells^41^. As 2i could prevent spontaneous differentiation in ES cells but not that by forcible expression of cell surface syntaxin-4, it is conceivable that one of the key roles of 2i might be to prevent membrane translocation of syntaxin-4.

Whereas ES cells treated with 2i succumbed to syntaxin-4-triggered differentiation, morphological changes induced by the syntaxin-4 signal were blocked. Considering that syntaxin-4 led to inactivation of Akt, which negatively regulates GSK3β, a potent regulator of E-cadherin expression^42^,^43^, GSK3β might mediate syntaxin-4 signaling to induce the morphogenic effect but not differentiation. GSK3β has been reported to play key roles in the perturbation of clonogenic activity in ES cells^44^; however, activation of downstream elements equivalently depend on the phosphorylation and subcellular localization of this protein^16^. Thus, it is not surprising that the syntaxin-4 signaling pathway inactivates PI3K/Akt signaling, leading to the activation of GSK3β for morphological changes, but not differentiation initiation.

An E- to P-cadherin switch was identified as a key element to mediate the morphologic function of syntaxin-4. Given that the expression of E-cadherin is strictly regulated by PI3K/Akt in ES cells^43^, the downregulation of E-cadherin could be attributed to the syntaxin-4-induced dephosphorylation of PI3K/Akt. It is noteworthy that the inactivation of PI3K/Akt signaling by cell surface syntaxin-4 occurred even in the presence of LIF, suggesting that the effect of syntaxin-4 might be dominant over LIF-triggered PI3K/Akt activation. Moreover, as GSK3β is a component of Wnt signaling, its subsequent activation may also affect the fate of β-catenin^45^. Thus, the elimination of E-cadherin might be accompanied by changes in subcellular localization or stability of β-catenin, a potent transcriptional activator for P-cadherin^46^. Transcriptome analyses did not detect the upregulation of other transcription factors that regulate P-cadherin such as p63 and C/EBPβ^47^,^48^. All of the classical cadherins, including E-, P-, and N-cadherin, play central roles in intercellular adhesion systems^49^,^50^; however, they exert different functions such as contact inhibition of locomotion via affecting the spatial distribution and activation of small GTPases^51^,^52^. A recent report demonstrated the regulation of cell migration by Cdc42, which is highly dependent on P-cadherin, but not E-cadherin^53^. In addition, P-cadherin fails to localize to the cell–cell contact sites and causes invasive cell behaviors in several aggressive cell types^54^. This study detected P-cadherin localization to the cell membrane rather than to cell–cell contact sites, suggesting that the P-cadherin-dependent morphological changes involved a function other than that in intercellular adhesion. Alternatively, certain cell surface molecules that can associate with P-cadherin but not E-cadherin—for example, integrin α6β4^18^—could propagate signals for morphological changes.

In addition to morphological changes, the syntaxin-4-triggered inactivation of PI3K/Akt signaling is a plausible differentiation cue, as this signaling pathway reportedly mediates the upregulation of genes involved in self-renewal^43^, retention of transcription factors for maintenance of stemness^23^, and inhibition of Wnt/MAPK- induced differentiation signals in ES cells^1^,^55^. However, ES cells failed to upregulate the differentiation marker *brachyury* in response to an Akt inhibitor, despite a significant downregulation of the stemness factor *zscan4*. Thus, whereas the activation of PI3K/Akt might be sufficient for maintenance of ES cell pluripotency^56^, its specific inactivation might be necessary but insufficient for activation of the differentiation program. Presumably, additional signaling pathways that could cooperate with PI3K/Akt signals to mediate differentiation in ES cells are propagated by cell surface syntaxin-4.

An important and contentious point is the causal link between morphological changes and differentiation in pluripotent ES cells. Although morphological changes and onset of differentiation are usually induced concurrently, an engineered substrate using recombinant E-cadherin caused a flattened morphology without differentiation^57^, and inhibition of GSK3β/MEK1/2 (2i-treatment) with forcible cell surface expression of syntaxin-4 resulted in the onset of differentiation without morphological changes. On the other hand, syntaxin-4-triggered inactivation of PI3K/Akt signaling subsequently downregulated *zscan4* during differentiation and severely affected cadherin-mediated cell adhesion systems involved in cell morphology. Accordingly, one might assume that these phenomena could be studied independently if signals downstream of PI3k/Akt are of interest. However, cell surface syntaxin-4 rapidly elicited mesodermal differentiation in ES and P19CL6 cells, whereas its sustained stimulation ultimately led to endodermal differentiation in F9 cells that are unable to differentiate into mesodermal lineages. This indicates that syntaxin-4-triggered changes in cell shape could activate a differentiation program for non-mesodermal lineages, albeit to a lesser extent. Thus, it is important to account for the fact that morphological changes and differentiation might have cause–effect relationships.

Although this study underlined the pernicious effect of syntaxin-4 signaling on the maintenance of ES cell stemness, the biological relevance to embryogenesis *in vivo* remains unknown. Disruption of *syntaxin-4* reportedly resulted in early embryonic lethality; however, this could be due to the functional ablation of cytoplasmic syntaxin-4 given its important role as a t-SNARE protein in intravesicular fusion^58^. We surmise that signals from cell surface syntaxin-4 might exert their functions at early developmental stages in embryogenesis, since EMT-like cell behavior accompanied by a cadherin switch has been proposed to be essential for gastrulation or neural crest delamination^52^,^59^,^60^. Although in preliminary experiments we detected extracellular syntaxin-4 in early-stage mouse embryos, its localization was not restricted to cells of the primitive streak or other specific regions. Moreover, attempts were made to generate transgenic mice expressing signal peptide- and T7-tagged syntaxin-4; however, no pups expressing the transgene product have been obtained to date. Whereas one pup from 62 manipulated-fertilized eggs was found to possess the transgene, T7-tagged syntaxin4 was not expressed, implying that extracellular syntaxin-4 severely disturbed normal embryogenesis. Careful analyses of successive developmental processes in fertilized eggs are necessary to clarify this issue.

## Methods

### Cell lines

The mouse ES cell line E14-Tg2a and that expressing the syntaxin-4 transgene (ES-STstx4) were cultured in GMEM (Wako) containing 10% FCS (Invitrogen), 1 mM glutamine (Wako), non-essential amino acid (Wako), 0.1 mM 2-mercaptoethanol (Wako), 1 mM pyruvate (Sigma) and LIF (Wako). To maintain stemness, inhibitors of GSK3β/MEK1/2 (2i) (CHIR99021/PD0325901; Invitrogen) dissolved in DMSO were added to the medium at a concentration of 2 μM. The mouse EC cell line F9 (ATCC CRL-1720) and its derivatives (F9-STstx4 and F9-Pcadherin) were maintained in DMEM/HamF12 medium (Wako) supplemented with 10% FCS. The P19CL6 cell line (RIKEN BRL RCB2318) and its derivative (P19-STstx4) were cultured in Alpha modified MEM (αMEM; Wako) with 5% FCS. To induce transgene expression, ES-STstx4, F9-STstx4, F9-Pcadherin, or P19-STstx4 was treated with 5 μg/ml Dox (Sigma-Aldrich) for two or three days. In some cultures, an inhibitor of PI3K (LY294002; Wako) was dissolved in DMSO and added at a concentration of 1.25 or 2.5 μM.

### Expression constructs and transfection

To construct the plasmid for tetracycline (Tet)/Dox-inducible expression of syntaxin-4 or P-cadherin, cDNA encoding syntaxin-4 with an N-terminal fusion of the IL2 signal peptide and T7 peptide tag^61^ or cDNA encoding P-cadherin (a generous gift from Dr. Takeich) was subcloned into the PiggyBac-TET transposon plasmid^34^,^62^,^63^. The plasmid harbored cDNA of the transgene and the neomycin-resistance gene connected by the IRES sequence. To generate cells with Dox/Tet-dependent expression of the transgene, one of these plasmids was transfected into cells, together with PB-CArtTA Adv and pCAG-Pbase^33^, using Lipofectamine 2000 (Life Technologies). To avoid a possible clonal artifact, a mixture of the clones that express the transgene product in response to Dox treatment (5 μg/ml) was used for experiments. These transfected cells included ES-STstx4 (ES cells with signal peptide/T7 tag-connected syntaxin-4), F9-STstx4 (F9 cells with signal peptide/T7 tag-connected syntaxin-4), F9-P-cad (F9 cells with P-cadherin), and P19-STstx4 (P19CL6 cells with signal peptide/T7 tag-connected syntaxin-4). To assess the ratio of cells that extrude syntaxin-4 at the cell surface, ES cells were transfected with an expression plasmid for T7-tagged syntaxin-4 (without a signal peptide, T7stx4)^61^ and stained with anti-T7 antibody before and after cell permeabilization.

### Antibodies and immunocytochemistry

Primary antibodies against T7-tag (Novagen), αSMA (Sigma-Aldrich), β-actin, (Sigma-Aldrich), Akt, and Akt phosphorylated at (Ser473) (CST Japan) were used in this study. Anti-E-cadherin monoclonal antibodies (ECCD1 for functional blocking and ECCD2 for immunodetection) and anti-P-cadherin monoclonal antibody PCD1 were generous gifts from Dr. Takeichi. Affinity-purified polyclonal antibodies against syntaxin-4 were prepared in a previous study^61^. HRP-, Alexa 488-, and Cy3- labeled secondary antibodies were purchased from Sigma-Aldrich, Invitrogen and GE healthcare. To detect cell surface expression of endogenous syntaxin-4, non-permeabilized cells were stained simultaneously for syntaxin-4 and the negative control β-actin, as described previously^61^. In brief, living cells on chamber slides were incubated with a mixture of affinity-purified rabbit anti-syntaxin-4 antibody and mouse anti-β-actin monoclonal antibody. After washing twice with Tris-buffered saline (TBS) followed by fixation with methanol (having an initial temperature of −20 °C) for 10 min, the cells were incubated with a mixture of Alexa488-labeled anti-rabbit IgG and Cy3-labeled anti-mouse IgG antibodies. For conventional immunocytochemistry, the cells were permeabilized with −20 °C methanol for 15 min, and stained with primary and Alexa488- or Cy3-labeled secondary antibodies, as described above. For some samples, cells were permeabilized with 0.1% triton X-100 instead of methanol, and F-actin was stained directly with Alexa 488-phalloidin (Invitrogen). Nuclei were counterstained with DAPI (Sigma). Stained cells were photographed with a confocal microscope system A1 (Nikon) or a fluorescence microscope AXIOSHOP (Zeiss) with a CCD camera VB-7010 (Keyence, Japan). To detect cell surface expression of transiently introduced T7-tagged syntaxin-4 (T7stx4) on a blot, cell surface proteins were labeled with NHS-biotin and lysed in buffer containing 1% Triton X-100 and 1% NP-40. The cell lysate was immunoprecipitated with anti-syntaxin-4 antibodies, and analyzed with HRP-labeled anti-T7 antibody (Novagen) or streptavidin (Sigma-Aldrich), as described previously^40^. Concurrently, biotin-labeled extracellular proteins in the cell lysate were retrieved with neutrAvidin-beads (Thermo Scientific) and analyzed with antibodies against syntaxin-4 or cytoplasmic β-actin.

### Reverse transcription quantitative RT-PCR (qRT-PCR)

Total RNA was extracted from ES-STstx4, F9-STstx4, F9-P-cad, and P19-STstx4 cells cultured with or without doxycycline was extracted using an RNeasy Mini kit (Qiagen) and reverse-transcribed with an RNA-PCR kit (Takara). To assess transgene expression, cDNA was amplified using Quick tag (Toyobo) and primer pairs for *T7-syntaxin-4* (5′- GGG GCG GCC GCA TGG CTA GCA TGA CTG GTG GAC-3′ and 5′-TTT TAG CTG CGC CCG GAC C-3′) and *gapdh* (5′-GGATTTGGCCGTATTGG-3′ and 5′-TCATGGATGACCTTGGC-3′). qRT-PCR was then carried out using FastStart Essential DNA Green Master on a LightCycler Nano system (Roche) according to the manufacturer’s protocol (PCR; 45 cycles). The primer pairs used in this study are listed in Supplementary Table S2. As the expression of both *gapdh* and β*-actin* was not changed in cells with and without exogenous syntaxin-4 (Supplementary Fig. S4), the mRNA expression of differentiation markers was normalized to that of β-actin. qRT-PCR was conducted in triplicate.

### Recombinant proteins

Recombinant forms of syntaxin-4 fragment F1 (Met1~Glu110), F2 (Ala111~Arg197), and GFP were generated as 6 X histidine-tagged forms and purified as described previously^32^. These proteins were dialyzed against PBS, filtrated, and added to cultures at a concentration of 50 μg/ml for three days.

### Analysis of cell behaviors upon cell surface expression of syntaxin-4

The morphology of cells cultured in the presence and absence of Dox for two (F9-STstx4 and F9-P-cad) or three (ES-STstx4 and P19-STstx4) days was analyzed. The number of cells extending more than three pseudopodia (10 μm or longer) was counted in ES-STstx4, F9-STstx4 or F9-P-cad cells. The area occupied by a cell was calculated in P19-STstx4 cells using the ImageJ software^64^.

### RNA-seq analysis

To identify differentially expressed (DE) genes in ES cells upon stimulation with extracellular syntaxin-4, comparative transcriptome analyses were performed at the Phyloinformatics Unit of RIKEN CLST. To exclude genes affected by Dox itself, cDNAs from ES-STstx4 and parent ES cells, with and without doxycycline for three days, were analyzed. The transcriptome analysis identified 138 DE genes with an absolute LogFC value ≥2 and a q-value <0.01. RNA-seq data sets are available at the Gene Expression Omnibus under accession number PRJDB4923.

### RNA interference of Zscan4 genes

To suppress the expression of Zscan4 genes in ES cells, oligonucleotide siRNAs for which clear knockdown effects have been demonstrated^22^ were obtained from Nippon Gene (Tokyo, Japan) (Supplementary Fig. S5). The mixture of the siRNAs (20 nM in total) was transfected into parental ES cells in 12-well plate well using Lipofectamine™ RNAiMAX Transfection Reagent (Invitrogen). The expression of *zscan4* and *p-cadherin* was analyzed on day 3 by qRT-PCR. As a control, universal negative control NEGD/NEGAS labeled with Hilyte 488 (20 nM) (Nippon Gene) was used; the transfection efficiency was more than 70% as judged by the fluorescence.

### Statistical analyses

Results are expressed as the mean ± SD of three independent experiments. Data were analyzed by a Student’s t-test or Mann–Whitney U-test, and a p-value of <0.05 was considered statistically significant.

## Additional Information

**How to cite this article**: Hagiwara-Chatani, N. *et al*. Membrane translocation of t-SNARE protein syntaxin-4 abrogates ground-state pluripotency in mouse embryonic stem cells. *Sci. Rep.* **7**, 39868; doi: 10.1038/srep39868 (2017).

**Publisher's note:** Springer Nature remains neutral with regard to jurisdictional claims in published maps and institutional affiliations.

## Supplementary Material

Supplementary Information
