# Supplementary material for: Membrane translocation of t-SNARE protein syntaxin-4 abrogates ground-state pluripotency in mouse embryonic stem cells
Source: Sci Rep. 2017 Jan 6;7:39868. doi: 10.1038/srep39868 (PMC5216394; doi:10.1038/srep39868)
Supplement: Supplementary Information [file srep39868-s1.pdf]

Supplementary information for:

Membrane translocation of t-SNARE protein syntaxin-4 abrogates ground-state pluripotency in mouse embryonic stem cells

by

Natsumi Hagiwara-Chatani[1], Kota Shirai[1], Takumi Kido[1], Tomoatsu Horigome[1], Akihiro Yasue[2], Naoki Adachi[1], Yohei Hirai[1]*

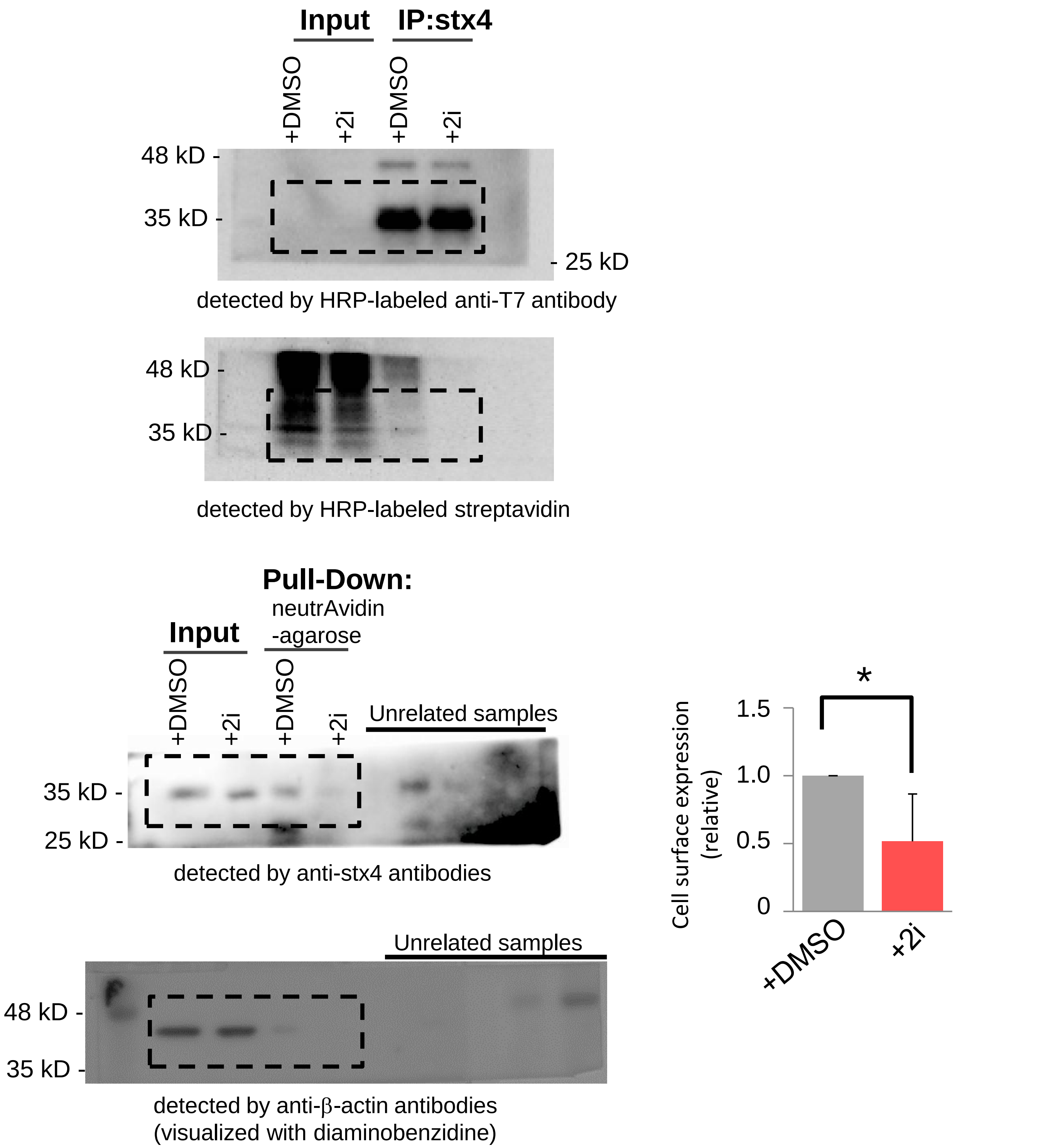

Original blot images of Fig. 1B (without image manipulation). Appropriate areas of the PVDF membrane, onto which protein bands had been transferred, were cut out and the target protein was detected. Dotted areas were cropped, processed within the guidelines, and used for the figure. Lower right, quantification of cell surface extrusion of endogenous syntaxin-4 from three independent experiments are shown. $*p < 0.05$.

Supplementary Fig. S1 (2/6)

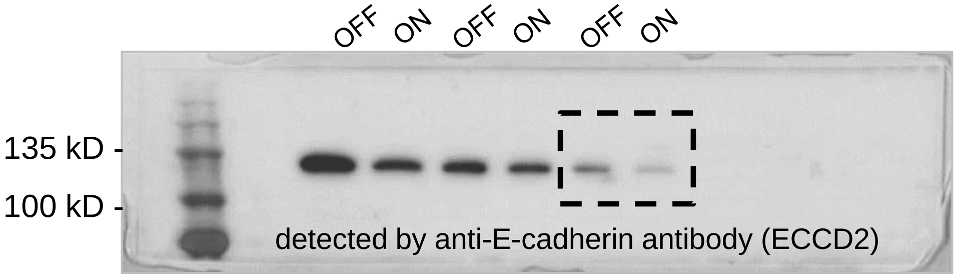

detected by anti-E-cadherin antibody (ECCD2)

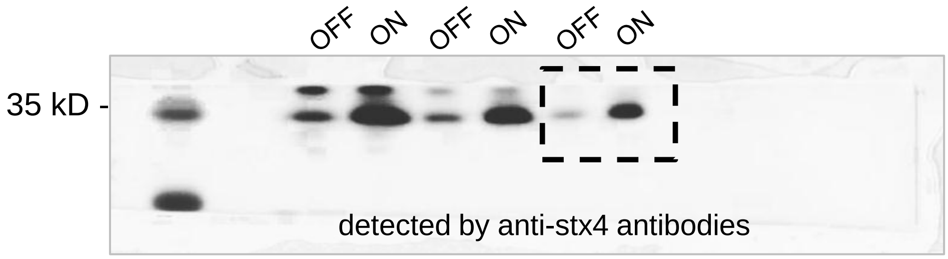

detected by anti-stx4 antibodies

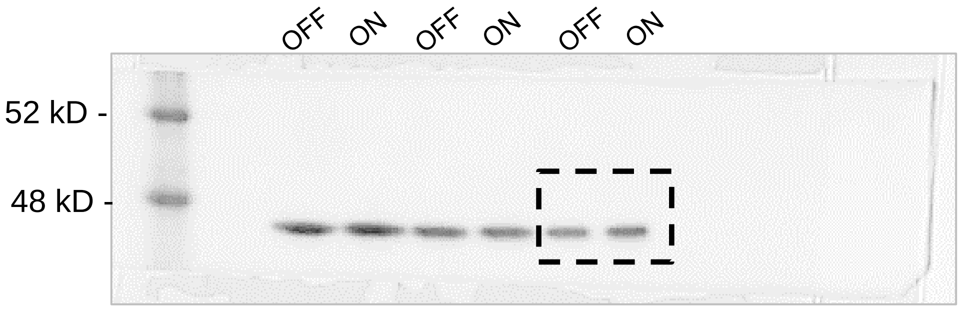

detected by anti-β-actin antibody

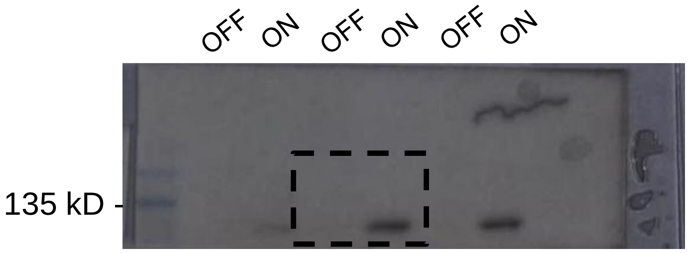

detected by anti-P-cadherin antibody (PCD1)

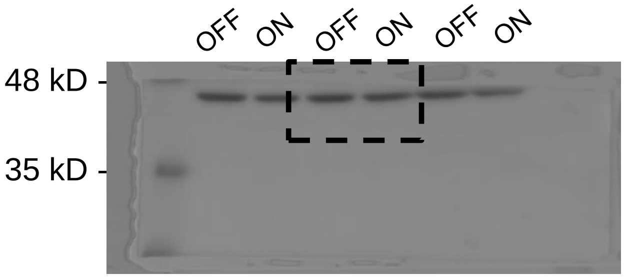

detected by anti-β-actin antibody

Original blot images of Fig. 2D (without image manipulation). Appropriate areas of the PVDF membrane, onto which protein bands had been transferred, were cut out and the target protein was detected. Dotted areas were cropped, processed within the guidelines, and used for the figure.

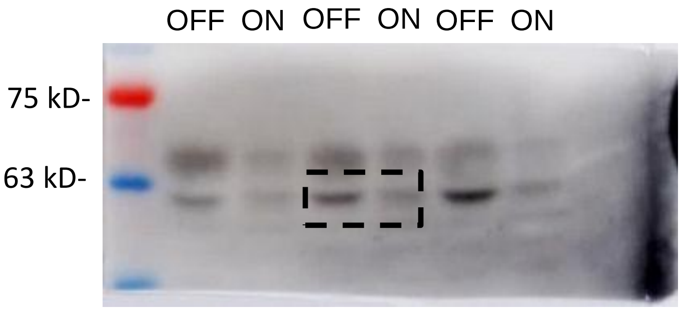

detected by anti-phosphor-Akt antibodies

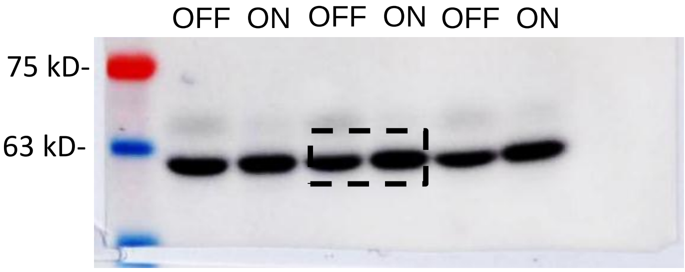

detected by anti-total-Akt antibodies

Original blot images of Fig. 3E (without image manipulation). Appropriate areas of the PVDF membrane, onto which protein bands had been transferred, were cut out and the target protein was detected.
Dotted areas were cropped, processed within the guidelines, and used for the figure.

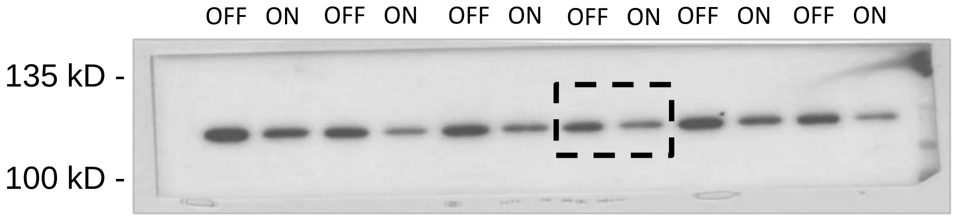

detected by anti-E-cadherin antibody (ECCD2)

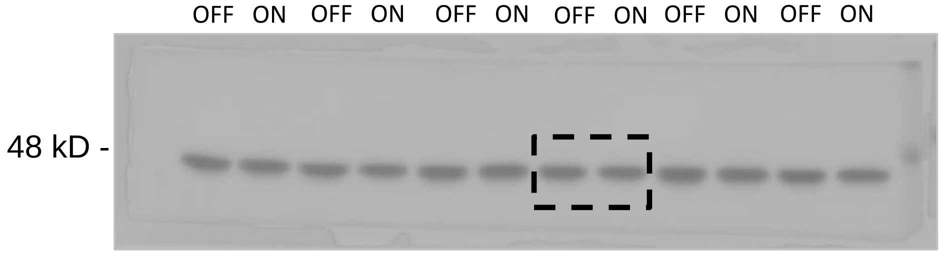

detected by anti-β-actin antibody

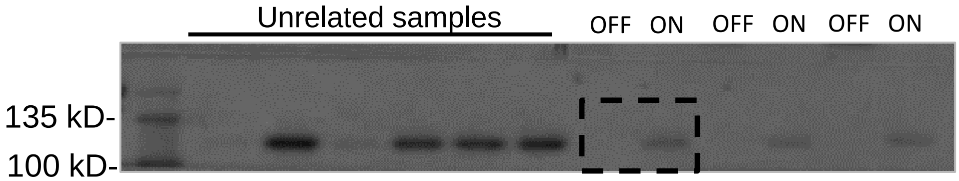

detected by anti-P-cadherin antibody (PCD1)

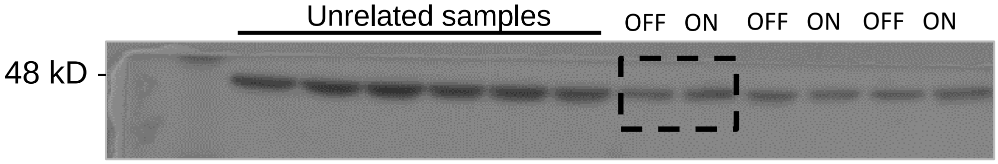

detected by anti-β-actin antibody

Original blot images of Fig. 5D (without image manipulation). Appropriate areas of the PVDF membrane, onto which protein bands had been transferred, were cut out and the target protein was detected.
Dotted areas were cropped, processed within the guidelines, and used for the figure.

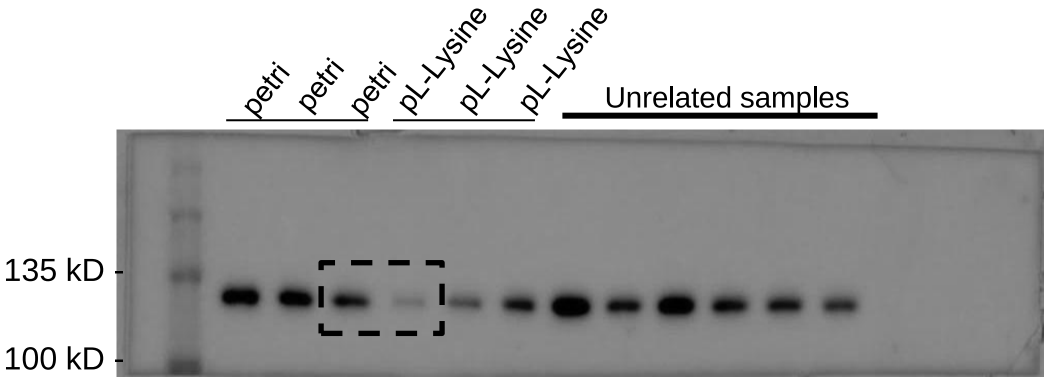

detected by anti-E-cadherin antibody (ECCD2)

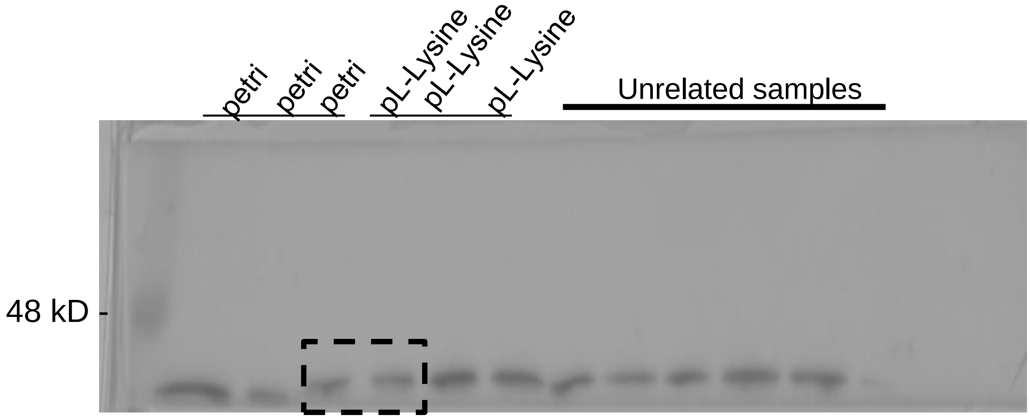

detected by anti-β-actin antibody

Original blot images of Fig. 6B (without image manipulation). Appropriate areas of the PVDF membrane, onto which protein bands had been transferred, were cut out and the target protein was detected. Dotted areas were cropped, processed within the guidelines, and used for the figure.

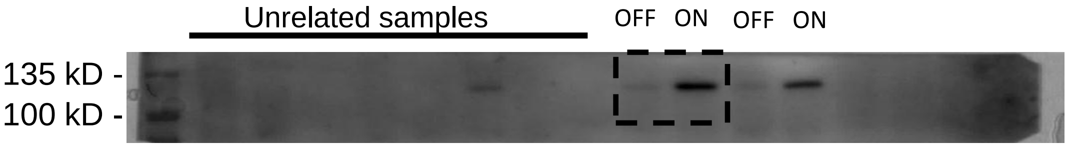

detected by anti-P-cadherin antibody (PCD1)

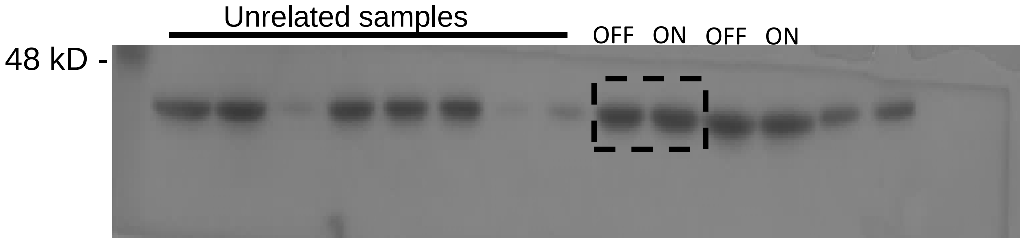

detected by anti-β-actin antibody

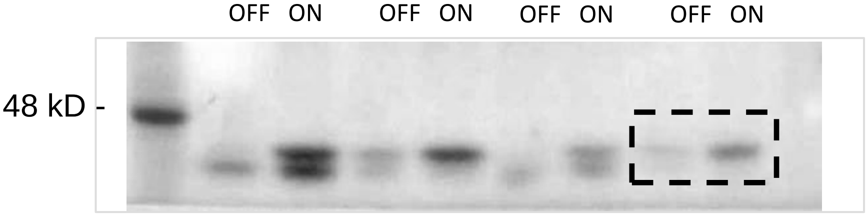

detected by anti-αSMA antibody

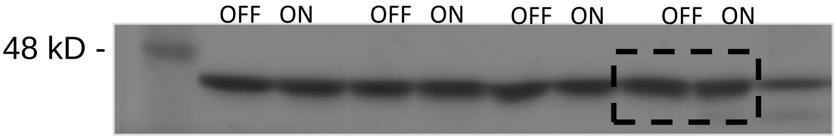

detected by anti-β-actin antibody

Original blot images of Fig. 7C (upper) and 7D (lower) (without image manipulation). Appropriate areas of the PVDF membrane, onto which protein bands had been transferred, were cut out and the target protein was detected. Dotted areas were cropped, processed within the guidelines, and used for the figure.

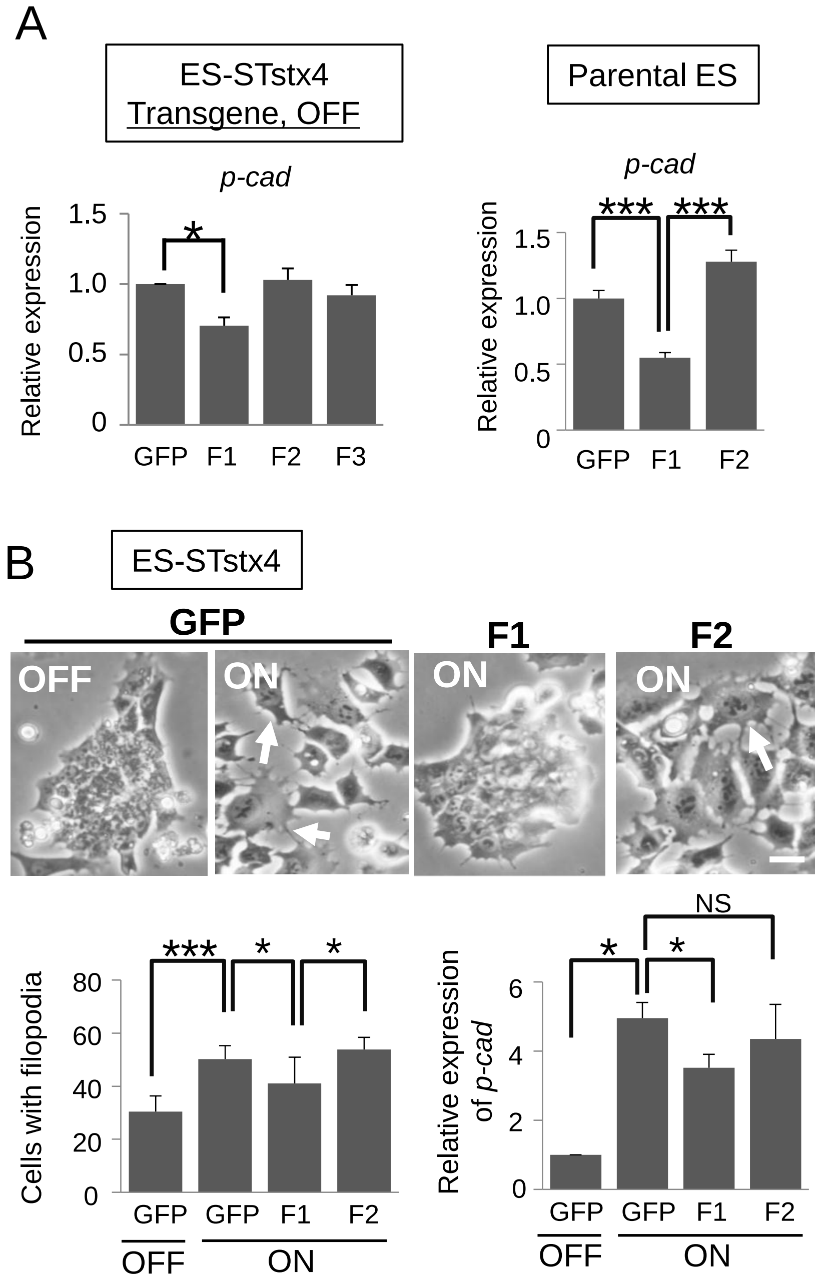

The antagonistic effect of syntaxin-4 fragments (A), Left, *p-cadherin* expression in ES-STstx4 cells without transgene expression. Cells were incubated with one of syntaxin-4 fragments or GFP control for three days. Recombinant F1 shows clear antagonistic effect, which is also the case in parental ES cells (right). N = 3. *$p < 0.05$, ***$p < 0.001$. (B), Upper images, phase-contrast images of ES-STstx4 cells (transgene, ON) incubated with recombinant F1, F2, or GFP control. Bar, 20 μm. Lower panels, quantification of cells with active filopodia (left; N = 6) and relative expression of *p-cadherin* (right, N = 4). *$p < 0.05$, **$p < 0.01$, ***$p < 0.001$. Cell surface expression of syntaxin-4 (ON) leads to morphological changes and upregulation of *p-cadherin*, both of which are blocked by the syntaxin-4 fragment F1.

Supplementary Table. S1 (1/3)

Up-regulated (42 Genes)

P-value	Q-value	logFC	Gene_Sym	Gene_ID
2.68E-25	2.71E-22	6.441901436	Pvalb	XLOC_007203
1.67E-08	0.00000148	6.378982017	Nebl	XLOC_011635
1.55E-07	0.00000991	5.757701258	Frrs1l	XLOC_014504
0.0000997	0.00234889	4.91686417	Wfdc1	XLOC_020241
0.00020709	0.00425449	4.784127639	Spink2	XLOC_015948
0.00036427	0.00651313	4.783773456	Rims1	XLOC_000682
0.00043172	0.00741562	4.637929669	4933422H20Rik	XLOC_003133
2.46E-08	0.00000207	4.356547654	Fut1	XLOC_018045
0.0000185	0.00057796	3.729680477	Edn3	XLOC_011535
0.00035223	0.00636155	3.727069834	Tsga10	XLOC_000722
8.85E-07	0.0000447	3.461856888	Duoxa1	XLOC_012226
4.09E-30	8.3E-27	3.454956441	T	XLOC_008204
0.00050787	0.0083368	3.271532311	5730405O15Rik	XLOC_022189
2.62E-09	2.73E-07	3.020340997	Col4a5	XLOC_022552
2.24E-08	0.00000192	2.819239058	Gad1	XLOC_010906
0.0000963	0.00227932	2.814505944	Gm3414	XLOC_015880
1.51E-47	1.75E-43	2.743397167	Stx4a	XLOC_018464
4.93E-35	1.64E-31	2.710025626	Shf	XLOC_012227
4.08E-17	1.5E-14	2.68816501	Frem2	XLOC_013206
0.00010698	0.00248674	2.651385185	Fer1l4	XLOC_012397
3.07E-07	0.0000178	2.633984759	Cxx1c	XLOC_022295
0.00000112	0.0000548	2.631951397	Cdx1	XLOC_009752
0.000017	0.00053887	2.577476868	Gm16023	XLOC_015025
0.00023762	0.00470864	2.576832407	Hmcn1	XLOC_001055
1.76E-08	0.00000155	2.571844654	Pcdh7	XLOC_015247
5.69E-10	6.99E-08	2.502587699	Thsd4	XLOC_021823
4.28E-22	2.93E-19	2.43136854	St3gal1	XLOC_007118
2.67E-17	1.02E-14	2.334411538	Madcam1	XLOC_001545
0.00000353	0.00014362	2.325212618	4930556M19Rik	XLOC_006631
2.17E-17	8.55E-15	2.325148438	Sp5	XLOC_010904
0.0000836	0.00201733	2.291380548	Fgf8	XLOC_010538
0.00000403	0.0001606	2.257592134	Col4a6	XLOC_023056
6.11E-11	9.36E-09	2.233708625	Cxcl12	XLOC_016819
0.0000002	0.0000123	2.200744747	Tnc	XLOC_014555
4.71E-21	2.96E-18	2.159019052	Chac1	XLOC_011169
0.00000169	0.000077	2.123771292	Plekhh3	XLOC_003878
0.0000283	0.00083244	2.077028614	Rcor1	XLOC_004479
5.71E-23	4.57E-20	2.070049006	Pck2	XLOC_005979
0.000026	0.00077332	2.066478069	Sat2	XLOC_002687
0.00040337	0.00701793	2.063773572	Arhgap5	XLOC_004192
4.08E-52	9.47E-48	2.049901227	Cdh3	XLOC_020182
0.00000134	0.0000641	2.041189264	Bpifb5	XLOC_011387

Down-regulated (96 Genes)

P-value	Q-value	logFC	Gene_Sym	Gene_ID
0.0000162	0.00051574	-2.045192342	Ces2e	XLOC_020145
0.00046709	0.00783342	-2.060269848	Cox7b2	XLOC_015920
1.5E-11	2.74E-09	-2.06093586	Slc13a5	XLOC_003575
0.00063112	0.0099254	-2.069395521	Tmprss11d	XLOC_015960
3.16E-12	6.63E-10	-2.070620985	Spink3	XLOC_009712
6.99E-15	1.98E-12	-2.11919187	Gpnmb	XLOC_016567
4.15E-09	4.19E-07	-2.121817674	Tmem92	XLOC_003748
0.00042281	0.00729261	-2.132696181	Mst1	XLOC_021379
0.00000543	0.00020529	-2.189481543	Gm12794	XLOC_013928
0.00021672	0.0044077	-2.192903602	Grin2a	XLOC_007817
1.33E-09	0.00000015	-2.20954754	Vdr	XLOC_007348
1.96E-17	7.97E-15	-2.21703053	Cyp1a1	XLOC_021140
0.0000034	0.00013917	-2.232918512	Ccr4	XLOC_022068
5.27E-23	4.37E-20	-2.235700067	Gpa33	XLOC_000496
0.00000228	0.00010019	-2.245182553	Mapk10	XLOC_016033
8.65E-07	0.0000438	-2.254647813	Spesp1	XLOC_021833
3.16E-16	1.01E-13	-2.295469837	Fbp2	XLOC_005567
4.78E-08	0.00000355	-2.348389077	Gm2016	XLOC_004340
0.0000469	0.00125862	-2.36029789	Ctrl	XLOC_020697
0.0000693	0.00174267	-2.360350273	Hist3h2a	XLOC_002598
0.00019785	0.00412442	-2.366275168	Cml2	XLOC_017295
0.0000105	0.00036549	-2.391585229	Gm11544	XLOC_003749
0.0000063	0.00023315	-2.400233761	Zscan4e	XLOC_018693
6.78E-09	6.38E-07	-2.405978803	D030018L15Rik	XLOC_007336
0.000024	0.00072132	-2.412144203	Wdr20b	XLOC_004221
4.28E-30	8.3E-27	-2.415227709	Gm7102	XLOC_010593
0.00048738	0.00810348	-2.427818194	Krt5	XLOC_007415
0.00053241	0.00862386	-2.456332486	Nckap5	XLOC_000972
0.0005324	0.00862386	-2.456335165	Krt15	XLOC_003852
0.00053235	0.00862386	-2.456349832	AI847159	XLOC_012262
1.08E-13	2.68E-11	-2.495241359	Usp17le	XLOC_019383
0.00000854	0.00030421	-2.510470321	Ndnf	XLOC_016631
0.0000173	0.00054724	-2.511268196	Spink10	XLOC_009523
0.00029671	0.00558443	-2.532795091	Csf1r	XLOC_009515
0.0004492	0.00765491	-2.533664955	Dnajb13	XLOC_019296
1.07E-16	3.64E-14	-2.542263112	Gm8300	XLOC_004335
2.3E-32	5.93E-29	-2.566794203	Dusp27	XLOC_001128
8.02E-12	1.55E-09	-2.601310778	Usp17la	XLOC_018303
0.00050022	0.00825203	-2.605538775	Cyp11a1	XLOC_021144
0.0000909	0.00217432	-2.674528102	Tm4sf1	XLOC_013213
8.94E-27	1.09E-23	-2.720357356	Gm2022	XLOC_004341
0.0000391	0.00108262	-2.755101271	Gm5622	XLOC_005944
0.00000032	0.0000184	-2.770381603	Prmt8	XLOC_017485
7.05E-20	4E-17	-2.779433838	1700030L20Rik	XLOC_013600
6.19E-14	1.6E-11	-2.781311721	Gm5039	XLOC_004760
0.00061814	0.00976089	-2.795392758	Cd96	XLOC_008017
0.00008	0.00194813	-2.805727893	Tesc	XLOC_015487
0.0002569	0.0050042	-2.80923818	Xirp2	XLOC_010888

P-value	Q-value	logFC	Gene_Sym	Gene_ID
8.75E-08	0.00000603	-2.847680419	Gm1995	XLOC_004339
0.0000229	0.00069536	-2.863481368	Ctxn3	XLOC_009496
2.12E-36	8.21E-33	-2.873074653	Dcdc2c	XLOC_004573
0.00021871	0.0044399	-2.88228013	BB287469	XLOC_004337
0.00032465	0.0060176	-2.912221171	AI662270	XLOC_002863
7.46E-14	1.88E-11	-2.920541889	Gm4971	XLOC_019176
1.24E-17	5.34E-15	-2.965025919	Zscan4a	XLOC_018690
0.00000442	0.00017278	-2.977802365	Gm13119	XLOC_014241
3.11E-23	2.78E-20	-2.984901882	D17Ertd648e	XLOC_008774
6.06E-10	7.41E-08	-3.104147993	Gm2027	XLOC_004182
3.9E-46	3.02E-42	-3.122619226	Gm20767	XLOC_005282
0.00000552	0.00020807	-3.135977495	Gm13128	XLOC_014240
2.27E-08	0.00000193	-3.144462691	Fam159b	XLOC_005717
3.07E-26	3.57E-23	-3.176211149	Zscan4c	XLOC_017668
2.56E-07	0.0000152	-3.215734392	Tdpoz4	XLOC_012842
7.82E-28	1.3E-24	-3.228172572	AF067061	XLOC_005283
4.38E-19	2.19E-16	-3.230645622	Gm5662	XLOC_004758
3.09E-19	1.59E-16	-3.252258567	B020031M17Rik	XLOC_005755
0.00063188	0.00993056	-3.292229707	Trim43a	XLOC_021282
2.36E-19	1.25E-16	-3.342710106	Zscan4f	XLOC_017669
0.00023327	0.00464629	-3.396578899	Pramel6	XLOC_011007
1.85E-08	0.00000161	-3.401444404	Usp17lc	XLOC_018272
2.89E-34	8.39E-31	-3.412070659	Gm8994	XLOC_016945
0.00000136	0.0000649	-3.42872639	Cdhr2	XLOC_005090
2.94E-18	1.31E-15	-3.451114743	Zscan4d	XLOC_018692
0.00000802	0.00028902	-3.480606389	Cyp26b1	XLOC_017281
0.00011942	0.0027295	-3.493623914	Gm13109	XLOC_014944
0.0000611	0.00156284	-3.584555422	Usp17ld	XLOC_019337
0.0000312	0.0009057	-3.67007993	Ube2dnl1	XLOC_022491
1.56E-14	4.2E-12	-3.680689609	Tcstv3	XLOC_005285
2.99E-12	6.32E-10	-3.75088191	Cyp2b23	XLOC_018879
1.44E-07	0.00000926	-3.782138646	BC147527	XLOC_005284
2.18E-09	2.32E-07	-3.908072628	Gm4858	XLOC_012832
9.1E-08	0.00000624	-4.030260041	9330159F19Rik	XLOC_001362
0.0000167	0.0005305	-4.030877571	AF067063	XLOC_005753
0.00000107	0.0000524	-4.034966212	Iqub	XLOC_017044
1.81E-08	0.00000159	-4.377729415	Edn2	XLOC_014042
4.43E-19	2.19E-16	-4.723561445	Usp17lb	XLOC_019384
0.00048896	0.00812396	-4.792300963	Dpep3	XLOC_020701
0.00018905	0.00397638	-4.796045477	BC080695	XLOC_014232
0.00018903	0.00397638	-4.796063808	A430089I19Rik	XLOC_016009
0.0000903	0.00216313	-4.928822088	Zscan4b	XLOC_018691
0.00041638	0.00720649	-4.937617042	Gm13078	XLOC_014234
0.00023877	0.00472746	-5.042242675	Trim75	XLOC_020503
0.0000347	0.00098271	-5.16086524	Tdpoz3	XLOC_012843
0.0000208	0.000639	-5.16254066	Gm6890	XLOC_022378
0.0000111	0.00037847	-5.361193588	AU015228	XLOC_011247
0.00000483	0.00018667	-5.36358869	Gm21319	XLOC_004755

Supplementary Fig. S3

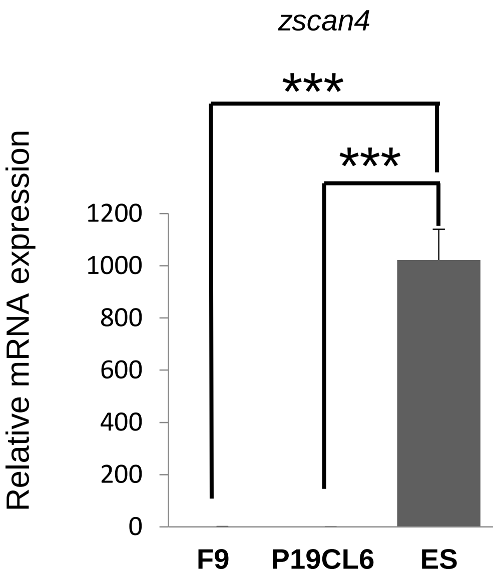

Expression of *zscan4* (*zscan4a, b, c, d* and *f*) in F9, P19CL6 and ES cells was analyzed by qRT-PCR. Primar pairs used are listed in Supplementary Table S2. N = 4, *** $p < 0.001$.

Primar pairs used for qRT-PCR analyses

Target	Forward	Reverse
E-cadherin	GCTCTCATCATCGCCACAG	GATGGGAGCGTTGTCATTG
P-cadherin	GCACTGCTGACCCTTCTACTG	GGGCTCTTTGACCTTCCTCT
Brachyury	CCACAAAGATGTAATGGAGGAAC	GAACAAGCCACCCCCATT
αSMA	CTCTCTTCCAGCCATCTTTCAT	TATAGGTGGTTTCGTGGATGC
MHC	GAAGGAGGAGGAGCTTCAGG	TCCTTGAAGCCTTTTCAGACTC
BMP4	GAGGAGTTTCCATCACGAAGA	GCTCTGCCGAGGAGATCA
Tuj1	CCCACTCCATGTGAGTCCA	GCAACATAAATACAGAGGTGGCTA
Nanog	TTCTTGCTTACAAGGGTCTGC	CAGGGCTGCCTTGAAGAG
Oct3/4	GTTGGAGAAGGTGGAACCAA	CTCCTTCTGCAGGGCTTTC
Slug	CATTGCCTTGTGTCTGCAAG	AGAAAGGCTTTTCCCCAGTG
Snail	CTTGTGTCTGCACGACCTGT	CAGGAGAATGGCTTCTCACC
Foxc2	GCAACCCAACAGCAAACTTTC	GACGGCGTAGCTCGATAGG
Vimentin	TGCGCCAGCAGTATGAAA	GCCTCAGAGAGGTCAGCAAA
Cofilin	TCCTTCTTCTCGTCCCAGTG	TCATTCACTGTAACTCCAGATGC
Zscan4	GACTGAACTATCTAACATCCTCAGCA	TTGCAACATTCTTCTCTCTTTGA
Gata4	GGAAGACACCCCAATCTCG	CATGGCCCCACAATTGAC
AFP	TGGATGTCAGGACAATCTGG	GCAGCTTTGCTTGGACAGT
Zeb1	ACCCCTTCAAGAACCGCTTT	CAATTGGCCACCACTGCTAA
Gapdh	TGACCACAGTCCATGCCATC	GACGGACACATTGGGGGTAG
β-actin	CCTCACCCTCCCAAAAGC	GTGGACTCAGGGCATGGA

Supplementary Fig. S4

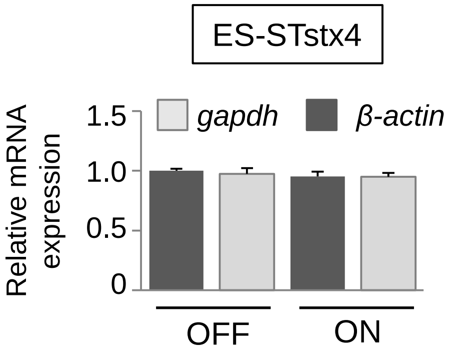

Relative expression of *gapdh* (□) and *β-actin* (■) in ES-STstx4 cells with (ON) and without (OFF) induction of cell surface syntaxin-4. Primar pairs used for the qRT-PCR analysis are listed in Supplementary Table. S2. N = 4

siRNAs used for gene-silencing of Zscan4.

siRNA Zscan4#1

5’guagcgauaugaggagauudTdT 3’
3’dTdTcaucgcuauacuccucuaa 5’

siRNA Zscan4#2

5’gaccaacaauuuagaguuudTdT 3’
3’dTdTcugguuguuaaaucucaaa 5’

siRNA Zscan4#3

5’caccaagugcucagcuaaadTdT
3’dTdTgugguucacgagucgauuu 5’

siRNA Zscan4#4

5’ gcugcaaagucucuggaagdTdT 3’
3’dTdTcgacguuucagagaccuuc 5’

Control siRNA

Hilyte 488 –labeled NEGS/NEGAS
(universal negative control)

Target sequences of the siRNAs.

Name of siRNA	Target position on cDNA (bp)	Target Sequences
siRNA Zscan4#1	514-532 (exon Ⅱ)	gtagcgatatgaggagatt
siRNA Zscan4#2	236-254 (exon Ⅱ)	gaccaacaatttagagttt
siRNA Zscan4#3	304-322 (exon Ⅱ)	caccaagtgctcagctaaa
siRNA Zscan4#4	362-380 (exon Ⅱ)	gctgcaaagtctctggaag
